# Prediction models for the complication incidence and survival rate of dental implants—a systematic review and critical appraisal

**DOI:** 10.1186/s40729-025-00590-1

**Published:** 2025-01-23

**Authors:** Yuanxi Zhu, Mi Du, Ping Li, Hongye Lu, An Li, Shulan Xu

**Affiliations:** 1 Center of Oral Implantology, Stomatological Hospital, School of Stomatology, Southern Medical University, Guangzhou, China; 2 School and Hospital of Stomatology, Cheeloo College of Medicine, Shandong University and Shandong Key Laboratory of Oral Tissue Regeneration, Shandong Engineering Laboratory for Dental Materials and Oral Tissue Regeneration, Shandong Provincial Clinical Research Center for Oral Diseases, Jinan, China; 3 Guangdong Engineering Research Center of Oral Restoration and Reconstruction, Affiliated Stomatology Hospital of Guangzhou Medical University, Guangdong, China; 4 Stomatology Hospital, School of Stomatology, Zhejiang University School of Medicine, Dental Biomaterials and Devices for Zhejiang Provincial Engineering Research Center, Zhejiang Provincial Clinical Research Center for Oral Diseases, Key Laboratory of Oral Biomedical Research of Zhejiang Province, Cancer Center of Zhejiang University, Hangzhou, China; 5 Department of Periodontology, Stomatological Hospital, School of Stomatology, Southern Medical University, Guangzhou, China

**Keywords:** Peri-implant disease, Predictive factor, Implantology, Prediction model, Systematic review

## Abstract

**Purpose:**

This systematic review aims to assess the performance, methodological quality and reporting transparency in prediction models for the dental implant’s complications and survival rates.

**Methods:**

A literature search was conducted in PubMed, Web of Science, and Embase databases. Peer-reviewed studies that developed prediction models for dental implant’s complications and survival rate were included. Two reviewers independently evaluated the risk of bias and reporting quality using the PROBAST and TRIPOD guidelines. The performance of the models were also compared in this study. The review followed the PRISMA guidelines and was registered with PROSPERO (CRD42019122274).

**Results:**

The initial screening yielded 1769 publications, from which 14 studies featuring 43 models were selected. Four of the 14 studies predicted peri-implantitis as the most common outcome. Three studies predicted the marginal bone loss, two predicted suppuration of peri-implant tissue. The remaining five models predicted the implant loss, osseointergration or other complication. Common predictors included implant position, length, patient age, and a history of periodontitis. Sixteen models showed good to excellent discrimination (AUROC >0.8), but only three had undergone external validation. A significant number of models lacked model presentation. Most studies had a high or unclear risk of bias, primarily due to methodological limitation. The included studies conformed to 18–27 TRIPOD checklist items.

**Conclusions:**

The current prediction models for dental implant complications and survival rate have limited methodological quality and external validity. There is a need for enhanced reliability, generalizability, and clinical applicability in future models.

**Supplementary Information:**

The online version contains supplementary material available at 10.1186/s40729-025-00590-1.

## Background

Dental implants have gained widespread popularity for partially or fully edentulous cases due to satisfactory therapeutic effects.About 3 million US citizens have dental implants and this number is rapidly growing by 500,000 annually [1]. Prevalence of dental implants of U.S. adult population projected to as high as 23% in the least by 2026 [2]. Despite the flourishing amount, dental implants’ complications and failure exert a heavy disease burden and pose a formidable challenge to dentists. Incidence of early implant failures, defined as the inability of tissue to establish osseointegration which were considered to occur during the early healing phase after implant surgery, is reported to be 5.6% [3–5]. According to a systematic review [6], implant supported fixed partial dentures without any biologic or technical complications were encountered in only 61.3% of patients after 5 years. On the one hand, with the improvement of implant technology and the updating of materials, the incidence of hardware complications has decreased significantly [7]. However, the overall incidence of hardware complications is still at a high level, with a 5-year incidence of 16.3–53.2% [7]. On the other hand, the biological complications of dental implants mainly consist of peri-implant mucositis and peri-implantitis, characterized by peri-implant bleeding, suppuration, bone loss, and implant loosening [8]. Those are common finding. According to a study, only 1.9 and 3.8% patients exhibited peri-implant health and clinical stable respectivly, while the remaining individuals presented with varying degrees of peri-implant disease [9]. The highest documented prevalence of peri-implantitis on implant level was 85.0% according to a systematic review [10]. The insidious onset and asymptomatic cases of peri-implant diseases make them easily overlooked, leading to an underdiagnosis. Though some biomaterial was studied to managing peri-implantitis [1], most patients with peri-implant diseases are at an advanced stage at diagnosis, with limited therapeutic effects. Therefore, prevention and early detection of high-risk patients are critical. Clinical prediction models are tools that predict health outcomes either at present (diagnostic) or in the future (prognostic) [11]. It was developed to accurately identify potential patients with unfavorable disease outcomes for appropriate early interventions [12].

Modern dentistry is moving toward precision medicine, with personalized treatment strategies in oral implantology tailored to each patient’s needs and informed clinical decisions using structured tools (*e.g.*, risk assessment tools or prediction models) [13, 14]. Several prediction modeling studies have recently been undertaken to predict the onset and progression of peri-implant complications [15, 16]. In addition, microbiome-based machine-learning models may be promising for predicting peri-implant inflammatory conditions [17–19]. Despite the predicting ability, examination of the risk of bias and reporting transparency are also important [20, 21]. Notably, a previous systematic review reported the methodological and reporting issues of prediction modeling studies for the incidence of periodontitis [22]. To the best of our knowledge, no systematic review has comprehensively mapped current progress toward the clinical application of prediction models in oral implantology.

This systematic review aimed to summarize all currently available prediction models to determine dental implants’ complication and survival rates, appraise model quality, and compare model performances.

## Methods

### Search strategy

This review was framed based on the Preferred Reporting Items for Systematic Reviews and Meta-Analyses (PRISMA) statement (Table *S*1) and was pre-registered on PROSPERO (No. CRD42023396454). Three electronic databases (PubMed, EMBASE, and Web of Science) were searched to identify studies on prediction models for dental implant survival, failure, and complications. The search term consisted of three concepts: (1) dental implant, (2) prediction model, and (3) longitudinal studies. Table *S*2 presents the complete list of search terms. Additionally, we thoroughly searched the bibliography of the retrieved full-text articles to identify any potential studies that might have been missed, using a method known as the “snowball process” as previously described [23].

### Eligibility criteria

The predictors included in this study encompassed sociodemographic, healthy lifestyle, systemic diseases, psychological, oral health-related, tooth-related, implant-related, and prosthesis-related factors. We included studies describing the development, validation, or assessment of models for predicting the outcomes of dental implants.

PICO for the included studies: (1) Population: Adult patients who have received dental implants, with a focus on those experiencing varying degrees of implant complication and related outcomes. (2) Intervention: The development of a predictive model aimed at forecasting the survival or complications of dental implants. (3) Comparison: The newly developed model will be compared to current predictive models to determine its comparative accuracy and clinical utility. (4) Outcome: Performance (AUROC, sensitivity, specificity, etc.), risk of bias and reporting quality of each prediction model study.

Studies meeting the following criteria were included: (1) patient cohort studies with sufficient sample size to test the hypothesis under investigation; (2) the predicted outcomes of dental implant therapy, including but not limited to implant survival, incidence, progression of implant complications, and other implant conditions; (3) the model containing at least two risk factors as predictors; (4) longitudinal study design.

Studies were excluded if: (1) they did not have original data such as review articles, meta-analyses, expert views, editorials, or comments, (2) the models included a single predictor, test, or marker, (3) they had no probabilistic samples, (4) they were published in the non peer-reviewed journals, (5) they did not provide clear predictive outcomes and the predictors were excluded, (6) they did not report accurate values (*e.g.*, the area under the receiver operating characteristic curve [AUROC], positive predictive value [PV+], negative predictive value [PV−], accuracy, sensitivity, specificity).

### Screening process

**Fig. 1:**
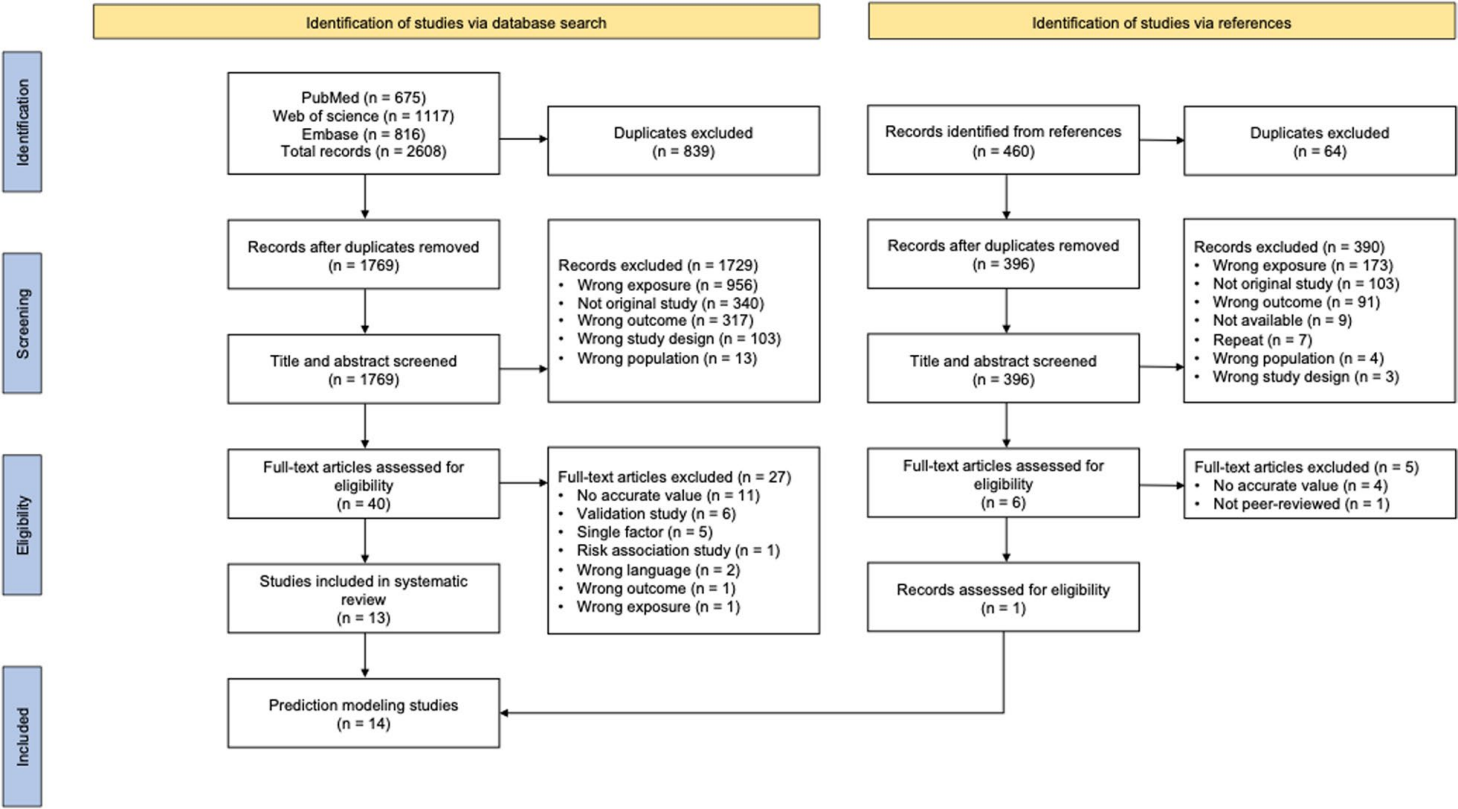
PRISMA flow diagram (Preferred Reporting Items for Systematic Reviews and Meta-analyses) of literature searching and selection

Two independent reviewers (YZ and AL) assessed the titles and abstracts in parallel and selected the articles based on the inclusion criteria. Two reviewers compared the selected articles after the initial screening. A referee (MD) was called to reach a consensus in case of any conflict. Subsequently, the full texts of the selected articles were read, and eligible studies were included. The references of these articles were screened to ensure no studies were overlooked. Figure 1 presents the study selection process in a flowchart.

### Data extraction

A standardized data extraction form was used to extract relevant information based on the general guidelines of the Critical Appraisal and Data Extraction for Systematic Reviews of Prediction Modelling Studies (CHARMS) checklist [24]. The extracted study characteristics included author/publication year, setting/context, study type, participant characteristics (mean age at baseline, sex, inclusion/exclusion criteria), sample size, the outcomes to be predicted, outcome definitions, and predictors. Concerning model development, information such as the model type, handling of missing values, candidate predictor selection, and final predictor selection were extracted.

### Predictors

The frequency of the final included predictors was calculated at the model level and the study level respectively. If multiple models are constructed in a study, the calculation method was to take the complement of all predictors of each model as the final variables included for the study level. For studies that did not report specific predictors, we included significant variables in the analysis. Identical variables with different names will be treated as the same item.

### Evaluation of prediction models

Information on discrimination, calibration, and validation was extracted to analyze the predictive performance of the included models. The discrimination ability of the model was assessed using AUROC, sensitivity, specificity, PV+ , PV−, and accuracy. 0.5 ≤ AUROC < 0.6 were considered to indicate poor discrimination, with 0.6 ≤ AUROC < 0.7, 0.7 ≤ AUROC < 0.8, 0.8 ≤ AUROC < 0.9, and AUROC ≥ 0.9 indicating moderate, good, very good, and excellent discrimination, respectively [25]. Sensitivity refers to the capability of the models to identify the individual with disease condition, while specificity refers to their ability to identify individuals without disease condition [26]. A positive predictive value indicates the probability that an individual identified by the model as having a disease truly have that disease condition [27]. Accuracy refers to the fraction of cases for which the model is correct [28]. Validation ability data, such as cross-validation and calibration ability data, were also extracted.

### Risk of bias assessment and reporting

Two independent reviewers (MD and YZ) used the PROBAST (Prediction Model Risk of Bias Assessment Tool) [29] to assess the risk of bias that may occur in terms of four domains: participants, predictors, outcomes and analysis. In case of conflict, a referee (AL) was called to help reach a consensus. The risk of bias was categorized as ‘low’, ‘moderate’ and ‘high’ levels.

### Transparency assessment

Incomplete reporting of studies is strongly discouraged to facilitate future efforts for validation, updating, recalibration, and guiding clinical practice. Transparency reporting was assessed with the TRIPOD (Transparent Reporting of a Multivariable Prediction Model for Individual Prognosis or Diagnosis) tool by two independent reviewers (MD and YZ). A referee (AL) was called to reach a consensus in case of conflict [20].

### Statistical analysis

We used medians for continuous variables and counts and percentages for categorical variables to describe the characteristics of all the included models.

## Results

### Selection process

The titles and abstracts of 1769 unique records were screened, and 40 studies meeting the inclusion criteria were included. After examining the full texts, 13 prediction modeling studies were identified. Figure 1 presents the whole screening process and the reasons for exclusion. Excluded reasons for full text screening of studies via database search and references were shown in supplementary file (Table S3–4). Common reasons for the exclusion during the title and abstract screening process included wrong exposure (no risk assessment tool and prediction model were found) and not being an original study. Common reasons for excluding full-text assessment were no accurate value (*i.e.*, AUROC, sensitivity, specificity, PV+, PV−, accuracy) and validation study. Then, 13 prediction modeling studies were included. The references of these 13 studies were also screened, leading to the inclusion of one additional study. Finally, 43 individual prediction models from 14 studies were included.

### Study designs and study populations

Of the 14 studies, 12 studies were retrospective cohort studies while the remaining two were prospective cohort studies (Table 1). The follow-up duration of these studies ranged from six months to more than five years. The sample size in the 14 studies ranged from 12 to 603 at the patient level, and 24 to 1206 at the implant level. The mean age of the participants ranged from 44.5 to 68.8 years. The percentage of female participants ranged from 50 to 75.9%.

**Table 1:** Characteristics of 14 prediction modelling studies

Study		Setting/Contex	Source of data	Participants characteristic	Follow-up (Year)	Outcomes to be predicted	Sample Size	Predictors (n)	Outcome definitors/classifications
Author/Year	Model								
Papantonopoulos et al. [34]	Predictive model for peri-implantitis	Patras, Greece	Retrospective cohort	1. Age (years) 60.3 ± 9.9 (27–80) 2. Gender (male/female) 44.8%/55.2%	At least 2 years, 7.1 ± 4.1 years on average	Patient mean peri-implant bone level (PMPIBL)*	94 patients with 340 implants	3	PMPIBL measured on radiographs at inter-proximal sites
Sampaio Fernandes et al. [38]	Prediction model for biological complications of implant overdentures	Northern region of Portugal	Retrospective cohort	1. Participants were aged between 50 and 86 years with an average of 68.8 2. 75.9% females 3. edentulous patients	At least 6 months	Biological complications of implant overdentures^	58 patients with 229 implants	5	Biological complications which include Peri-implant inflammatory signs (erythema, suppuration or fistula), Mobility, Pain, Peri-implant mucositis, Periimplantitis, Loss of dental implant
Papantonopoulos et al. [35]	Prediction model of individual implant bone levles	Patras, Greece	Retrospective cohort study	1. Participants age 61.9 ± 11.1 2. 51.4% women	At least 2 years, 7.4 ± 3.5 on averge	Individual implant bone levels (IIMBL) *	72 implant-treated subjects with 237 implants	6	An IIMBL was calculated as the mean of the two approximal measurements on radiographs
Zhang et al. [15]	Nomogram prediction model of peri-implantitis	Beijing, China	Prospective cohort study	1. The mean $\pm$ SD age was 44.5 $\pm$ 9.4 years 2. 50% male 3. Participants were diagnosed with severe periodontitis	1–5 years	Peri-implantitis^	100 patients with 214 implants	1. Stepwise model = 2 2. Full model = 4	• Presence of bleeding and and/or suppuration on gentle probing • Increased probing depth (PD) compared to previous examinations • Presence of bone loss beyond crestal bone level changes resulting from initial bone remodeling
Ha et al. [39]	Predictive model of dental implants	Korean Seoul	Retrospective cohort study	Not provided	1 year	Survival and complication of the implants^	53 patient and 59 cases	4–7 in each models	Survival of the implant: whether the implant was osseointegrated or not; Complications of the implant: any discomfort or defect despite of adequate function with osseointegration
Nobre et al. [37]	A prognostic model for ailing and failing implants due to peri-implant disease	Lisbon, Portugal	Retrospective cohort	1. Patients over 18 years with average age of 57.3 years 2. male: 97; female: 143 3. Patients with at least one ailing or failing implant	1 year	Implant status^	240 patients	5	Implant status: success, defined as the arrest of the disease, or failure defined as implant extraction, prevalence or re-incidence of peri-implant disease • Peri-implant disease: the presence of peri-implant pockets ≥5 mm, bleeding on probing and/or suppuration, with concurrent presence of marginal bone loss or clinical attachment loss ≥2 mm comparatively to the previous examination
Wang et al. [19]	Classification model with microbiota in identifying sites with or without suppuration	Beijing, China	Retrospective cohort	1. Patients with a mean age of 48 years 2. 41.7% male 3. Stage IV, grade C periodontitis 4. At least one implant diagnosed as peri-implant mucositis	2 years	Suppuration of peri-implant mucositis^	12 patients with 24 implants	12	The presence of suppuration (SUP) was assessed carefully using light forces
Zhang et al. [17]	Prediction model for marginal bone loss of dental implant in the mandible	Nanjing, China	Retrospective cohort	1. Patients aged above 18 2. Patients with mandible implant	A mean 20.95 ± 2.67 months of follow-up after functional loading	The occurrence of severe marginal bone loss (MBL) in of dental implant in mandible^#^	81 patients	NP	• Normal controls less than 2 mm MBL in the first year after fixed prosthesis, then less than 0.2 mm MBL per year • Severe MBL cases MBL level exceeding normal controls
Lu et al. [18]	A microbial prediction model for clinical suppuration	Beijing, China	Prospective cohort	1. Patients with a history of periodontitis and classified as Stage III 2. Mean age of 48.00 3. 41.7% male	2 years	Suppuration of peri-implant mucositis^	12 patients with 24 implants	11	Suppuration probing was measured from the mucosal margin to the bottom of the probeable pocket with a light force (approximately 0.25 N) at the mesial, buccal, distal and lingual surfaces of implants and presence or absence of bleeding/suppuration up to 30 s after probing was assessed
Mameno et al. [31]	Machine learninig predictive model for peri‑implantitis	Osaka, Japan	Retrospective cohort	1. Mean age of 67.06 in PI-group and 65.77 in Non-PI-group 2. 66.3% women	At least 4 years	Peri-implantitis^	254 implants	NP	Peri-implantitis was defined as the presence of bleeding on probing and/or suppuration in the follow-up period and the presence of more than 1 mm of bone resorption from the baseline measurement
Zhang et al. [33]	Nomogram model to predict the risk of peri-impalntitis in patients with diabetes mellitus	Dalian, China	Retrospective cohort	1. Mean age of 44.9 ± 6.7 2. 66.3% women 3. Diganosed with diabetes	1 year	Peri-implantitis^	257 paitents	6	BOP, SOP, PPD ≥5 mm, radiographic marginal bone loss ≥2 mm
Huang et al. [36]	Prediction model for dental implant loss	Chongqing, China	Retrospective cohort	1. Mean age of 57.0 in high risk gourp and 52.9 in low risk groups 2. 71.5%male in high risk group and 66.5% male in low risk group	5 year	Implant loss^	603 patients with 680 implants	1. Clinical model: 8 2. Dl model: Radiographic feature 3. Intergrated model: 9	5-year implant loss risk
Oh et al. [40]	Deep learning-based prediction model of osseointegration for dental implant	Daejeon, Korea	Retrospective cohort study	1. Age range, 21–78 years 2. 311 men, 269 women	3 years or more	Osseointegration^	580 patients (1206 dental implants)	Radiographic feature	The reverse torque test was performed to confirm successful osseointegration at the time of abutment connection
Rekawek et al. [32]	A web-based implant failure and peri-implantitis prediction model	Philadelphia, USA	Retrospective cohort study	Not provided	At least 5 years	Implant failure and peri-implantitis^	942 implants placed in 398 unique patients	50	Peri-implantitis was defined as radiographic evidence of changes in the crestal bone level and clinical evidence of bleeding on probing, with or without suppuration Implant failure was defined as implant mobility or a lack of osseointegration at a minimum of 6 months, or bone loss >1 mm per year, evaluated after implant placement

*NP* not provided

^*^Continuous outcome variables were left unaltered. #, Continuous outcome variables were discretized. ^, Not continuous outcome

### Outcome definition

Table 1 shows that four of the 14 modeling studies predicted peri-implantitis as the most common outcome. However, the definition of peri-implantitis varied. The definition of peri-implantitis in two models from Zhang’s study followed the consensus report of the 2017 World Workshop on the classification of periodontal and peri-implant diseases and conditions [15, 30], which included the presence of bleeding and/or suppuration on probing, increased probing depth (PD) compared to previous examinations, and bone loss beyond crestal bone level changes after initial bone remodeling. In Mameno’s study with three models, peri-implantitis was defined as the presence of bleeding on probing and/or suppuration in the follow-up period and the presence of >1 mm of bone resorption from the baseline measurement [31]. In Rekawek’s study, peri-implantitis was defined as radiographic evidence of changes in the crestal bone level and clinical evidence of bleeding on probing, with or without suppuration [32]. The definition of peri-implantitis in Zhang’s study included bleeding/ suppuration on probing, probing depth ≥5 mm, and radiographic marginal bone loss ≥2 mm [33].

In addition, two studies by Papantonopoulos and one study by Zhang predicted the marginal bone loss (MBL) of dental implants [17, 34, 35]. Three models in Huang’s study predicted implant loss [36]. Two studies reported models predicting the suppuration of peri-implant tissue [18, 19]. The remaining four models predicted the biological complications, survival and complication, peri-implant disease and osseointergration respectively [37–40]. Among the 14 modeling studies, one was a prognostic modeling study, while the rest were diagnostic modeling studies.

### Predictors

**Fig. 2:**
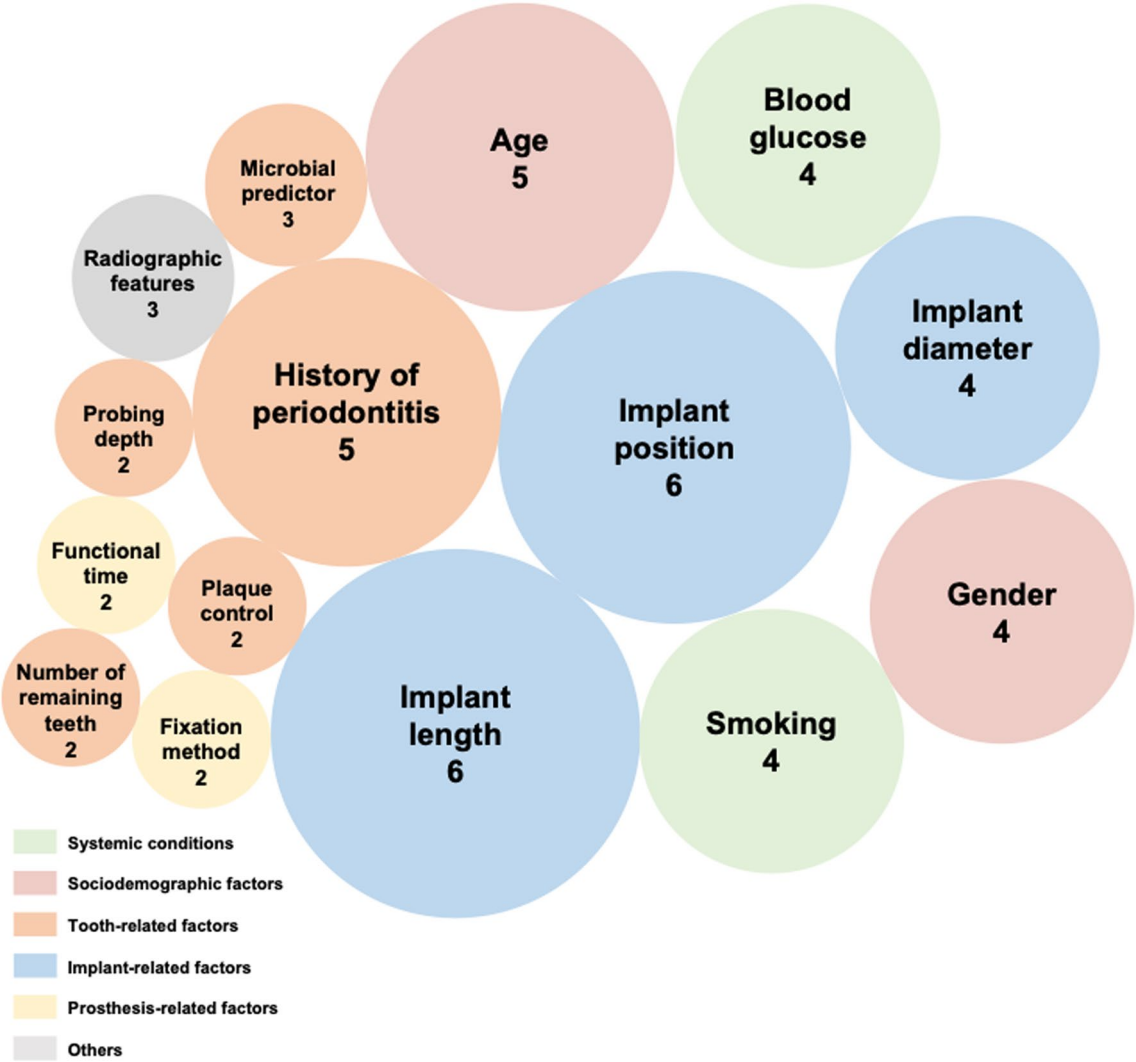
Frequency of risk predictors identified not less than twice in the final prediction models

The number of predictors in each included model ranged from two to 50, as shown in Fig. 2. The most frequently used predictors at the study level were implant positions and Implant length, followed by age and history of periodontitis. We identified 15 and 32 predictors used at least twice at models level and study level respectivley (Table S5–S6). The identified predictors were as follows: (1) Sociodemographic factors, including age and gender. (2) Behavior factors and systemic conditions, such as smoking habits and blood glucose. (3) Tooth-related factors, including history of periodontitis, microbial factors, probing depth, plaque control, number of remaining teeth. (4) Implant-related factors such as implant position, implant length, implant diameter. (5) Prosthesis-related factors, including the fixation method, functional time. (6) Other factors, such as radiographic features. Candidate predictors for each study were shown in supplement file (Table S7).

### Modeling method and model presentation

About a quarter of the 40 models (11/43) were logistic regression (LR) models, nearly half of the models (19/43) were machine learning (ML) models, 18.6% (8/43) were deep learning (DL) models and three were esemble models (LR combined with artificial intelligent [AI] method) (Table 2). For the other two models, one classification model was constructed with the canonical discriminate function based on the fisher method, while the other was based on generalized linear mixed models. Additionally, 12% (5/43) of the models were presented with full equations, 5% (2/43) were shown with nomograms. The other seven models were presented by saliency map, classification diagram, decision tree, box-plot, score chart, heat map and web-based application repectively. Nearly 70% of the models (29/43) were not presented in the studies.

**Table 2:** Model development, presentation, and interpretation of 14 prediction modeling studies

Study		Missing value	Variable selection	Model development	Model presentation	Model interpretation
Author/Year	Model					
Papantonopoulos et al. [34]	Predictive model for peri-implantitis	NP	PCA, forward feature selection	K-nearest neighbours regression, binary logistic regression	NP	Three factors used by the KNN method may provide reasonable prediction of PMPIBL
Sampaio Fernandes et al. [38]	Prediction model for biological complications of implant overdentures	NP	Univariate selection,.forward stepwise technique (pE = 0.15 and pR = 0.20). Bacteria were included though *p* values >0.20	Logistic regression model	g(xi) = -16.299 + 3.005 * MeanPD + 6.247 * Metalexposure + 3.224 * IL1B_alelle2 + 1.953 * Maxillary edentulousness—3.76 * F. nucleatum	The prediction model developed in this study could serve as a basis for further improved models that would assist clinicians in the daily diagnosis and treatment planning practice of oral rehabilitation with implant overdentures
Papantonopoulos et al. [35]	Prediction model of individual implant bone levles	NP	PCA, ES model, PSO-SVMs model	ES model, PSO-SVMs model	NP	ES and SVMs showed good results in predicting IIMBL respectively
Zhang et al. [15]	Nomogram prediction model of peri-implantitis	NP	Univariate and multivariate logistic regression analysis with generalized estimating equations. Stepwise model selection was performed by a backward step-down selection process (*P* < .05)	Logistic regression analyses with generalized estimating equations	The scoring stepwise model at T1 were as follows: 2.18059 * (posterior) + 1.13804 * PDT1 ≥6 mm% >10%). The stepwise model was illustrated by using nomogram The scoring full models at T1 were as follows: 0.20492 * (length ≥8 mm) + 1.64476 * (posterior) + 0.88415 * (PDT1 ≥6 mm% >10%) + 0.60729 * (diameter ≥5 mm)	The nomograms showed satisfactory predictive accuracy. It can be used to estimate the risk of peri-implantitis in treated periodontitis patients in Chinese
Ha et al. [39]	Predictive model of dental implants	Cases that lacked sufficient information for evaluation were excluded	RF model, SVM model	RF model, SVM model	The RF models were shown in Figs. 1 and 2	NP
Nobre et al. [37]	A prognostic model for ailing and failing implants due to peri-implant disease	Patient with medical records incomplete/missing; patients were excluded	Univariate logistic regression analysis, multivariable logistic regression analysis	Logistic regression model	The final model was presented in a score chart in Fig. 3	A prognostic risk model for estimating the outcome of implants with peri-implant disease is available, with a good performance considering the c-statistic evaluation
Wang et al. [19]	Classification model with microbiota in identifying sites with or without suppuration	NP	16S rRNA gene sequencing, GLMM	Canonical Discriminate Function based on the Fisher method	Non-SUP group: = 3446 G1 + 48,621 G2 − 415,261 G3 + 981,726 G4 − 116,036 G5 − 106,877 G6 + 2161 G7 − 1731 G8 + 1858 G9 + 10,216 G10 − 912,588 G11 + 3,251,561 G12 − 404 SUP group: = 147,828 G1 + 134,620 G2 − 1,156,706 G3 + 2,704,652 G4 − 325,562 G5 − 297,696 G6 + 5899 G7 − 4999 G8 + 5019 G9 + 285,863 G10 − 2,537,674 G11 + 9,034,524 G12 − 3058	Classification model with microbiota has a high accuracy in distinguishing peri-implant mucositis sites with or without suppuration
Zhang et al. [17]	Prediction model for marginal bone loss of dental implant in the mandible	Patients with an incomplete periodontal follow-up examination and treatment record were exlcuded	PCA and correlation covariance matrices	SVM model, ANN model, RF model, logistic regression model with variable choice through backward elimination	NP	Our results showed a great performance of ML methods for predicting MBL, which can be considered as the feasible early warning for severe MBL
Lu et al. [18]	A microbial prediction model for clinical suppuration	Patients who lost to follow up were excluded	16S rRNA gene sequencing, GLMM	GLMM	Figure 4b	Microbiota at the early stage could predict subsequent microbial dysbiosis and clinical suppuration
Mameno et al. [31]	Machine learninig predictive model for peri‑implantitis	Patients with missing data, insufficient data from dental record and unmeasurable radiographs was excluded	Logistic regression model, SVM model, RF model	Logistic regression model, SVM model, RF model	RF model classification diagram in Fig. 4	The model for predicting the onset of peri-implantitis by using the ML classification method is robust to the bias and collinearity of variables, and to clarify the relationships between risk indicators
Zhang et al. [33]	Nomogram model to predict the risk of peri-impalntitis in patients with diabetes mellitus	Only include patient with complete record	Single factor analysis, multivariable logistic regression analysis	Multivariable logistic regression analysis	The nomogram model was presented in Fig. 1	The nomogram model can intuitively and accurately predict the probability of peri-implantitis in diabetic patients
Huang et al. [36]	Prediction model for dental implant loss	Patients with incomplete information or CBCT was excluded	Clinical model: The variables are clinical features extracted from the EMR. Verified clinical features were used as the variables in the equation of the CM Deep learning model: CBCT images deep learning Intergrated modle: Combine variates in CM model and DL model	1. Clinical model: Conventional multi-variable logistic regression 2. Deep learning model: VGG16 3. Integrated model: The DL model’s last layer output for CBCT slices was averaged and added to the clinical model as a new parameter	1. Clincal model: Appendix Table S3: Variables in the equation of the CM 2. Deep learning model: Visualized with saliency maps in Fig. 4 3. Integrated model: Appendix Table S4: Variables in the equation of the IM	Both the DL model and the IM performed well in predicting implant fate within 5 years and thus may greatly facilitate implant practitioners in assessing preoperative risks
Oh et al. [40]	Deep learning-based prediction model of osseointegration for dental implant	NP	Deep learning	ResNet18 ResNet-34 ResNet-50 DenseNet-121 DenseNet-201 MobileNet-V2 MobileNet-V3	The visualization results of the best-accuracy model are presented in Fig. 4	Osseointegration of dental implants can be predicted to some extent through deep learning using plain radiography. This is expected to complement the evaluation methods of dental implant osseointegration that are currently widely used
Rekawek et al. [32]	A web-based implant failure and peri-implantitis prediction model	Patient with inadequate medical records were excluded K-nearest-neighbors imputation was employed to fill in missing values	Recursive feature elimination	Logistic regression model, RF model, SVM model Ensemble models	Web-Based Application	The incorporation of a machine learning model into a simple web-based interface that accurately predicts implant failure can become a vital resource and guide for patient care in the future

*NP* not provided, *PCA* Principal components analysis, *ES* ensemble selection, *SVM* Support vector machines, *RF* random forest, *GLMM* Generalized Linear Mixed Model, *ANN* Artificial neural network

### Predictive performance and validation

Table 3 presents the predictive performance and validation of the included prediction models. Three of the studies reported calibration data and two of them presented calibration plot. All studies reported discrimination. Among them, 35 models in ten studies reported AUROC, 27 models in six studies reported sensitivity (recall), 14 models in four studies reported specificity, 20 models in four studies reported PV+ (precision), seven models in two studies reported PV−, and 26 models in six studies reported accuracy.

**Table 3:** Model Performance and evaluation of 14 prediction modeling studies

Study		Predictive performance (Discrimination)						Validation	Calibration
Author/Year	Model	AUROC	Sen (Recall)	Spec	PV+ (Precision)	PV−	Accu		
Papantonopoulos et al. [34]	Predictive model for peri-implantitis	NP	0.526	0.707	NP	NP	NP	External validation (Root-mean-squared error of 0.141)	None
Sampaio Fernandes et al. [38]	Prediction model for biological complications of implant overdentures	0.95 (95%CI: 0.898–1.000)	NP	NP	NP	NP	NP	None	None
Papantonopoulos et al. [35]	Prediction model of individual implant bone levles	NP	1.ES model = 0.55 2.PSO-SVMs model = 0.62 3.10-fold cross-validation ES model = 0.38 4.10-fold cross-validation PSO-SVMs model = 0.47	1.ES model = 0.91 2.PSO-SVMs model = 0.85 3.10-fold cross-validation ES model = 0.90 4.10-fold cross-validation PSO-SVMs model = 0.79	NP	NP	NP	None	None
Zhang et al. [15]	Nomogram prediction model of peri-implantitis	1. Stepwise model = 0.794 2. Full model = 0.796	NP	NP	NP	NP	NP	None	None
Ha et al. [39]	Predictive model of dental implants	NP	NP	NP	NP	NP	1. RF model of implant survival = 0.93 2. RF model of implant complications = 0.64 3. SVM model of implant survival = 0.95 4. SVM model of implant complications = 0.75	None	None
Nobre et al. [37]	A prognostic model for ailing and failing implants due to peri-implant disease	0.763 (95%CI: 0.679–0.847)	NP	NP	NP	NP	NP	External validation (c-statistics = 0.709 (95%CI: 0.616–0.803))	Hosmer and Lemeshow test in validation set (*p* = 0.211)
Wang et al. [19]	Classification model with microbiota in identifying sites with or without suppuration	NP	NP	NP	NP	NP	1	None	None
Zhang et al. [17]	Prediction model for marginal bone loss of dental implant in the mandible	1. SVM model = 0.967 2. ANN model = 0.928 3. LR model = 0.906 4. RF model = 0.842	1. SVM model = 0.9167 2. ANN model = 0.9167 3. LR model = 0.9167 4. RF model = 0.7500	1. SVM model = 1.0000 2. ANN model = 0.9333 3. LR model = 0.9333 4. RF model = 0.8667	1. SVM model = 1.000 2. ANN model = 0.917 3. LR model = 0.917 4. RF model = 0.818	1. SVM model = 0.938 2. ANN model = 0.933 3. LR model = 0.933 4. RF model = 0.813	NP	None	None
Lu et al. [18]	A microbial prediction model for clinical suppuration	0.9	NP	NP	NP	NP	0.9	None	None
Mameno et al. [31]	Machine learninig predictive model for peri‑implantitis	SVM model = 0.64 RF model = 0.71 LR model = 0.63	RF model = 0.66	NP	RF model = 0.72	NP	RF model = 0.70	None	None
Zhang et al. [33]	Nomogram model to predict the risk of peri-impalntitis in patients with diabetes mellitus	0.867 (95%CI: 0.832–0.902)	NP	NP	NP	NP	NP	External validation (c-statistic 0.822)	Calibration curve
Huang et al. [36]	Prediction model for dental implant loss	1. Clinical model = 0.72(95%CI: 0.63–0.79) 2. Deep learning = 0.87(95%CI: 0.80–0.92) 3. Integrated model = 0.90(95%CI: 0.84–0.95)	NP	NP	1. Clinical model = 0.66 2. Deep learning = 0.81 3. Integrated model = 0.81	1. Clinical model = 0.64 2. Deep learning = 0.81 3. Integrated model = 0.82	NP	None	Calibration curve
Oh et al. [40]	Deep learning-based prediction model of osseointegration for dental implant	1. ResNet18model = 0.89 ± 0.04 2. ResNet-34 model = 0.91 ± 0.03 3. ResNet-50 model = 0.92 ± 0.02 4. DenseNet-121 model = 0.90 ± 0.03 5. DenseNet-201 model = 0.90 ± 0.03 6. MobileNet-V2 model = 0.90 ± 0.03 7. MobileNet-V3 model = 0.89 ± 0.03	1. ResNet18model = 0.811 2. ResNet-34 model = 0.832 3. ResNet-50 model = 0.817 4. DenseNet-121 model = 0.813 5. DenseNet-201 model = 0.827 6. MobileNet-V2 model = 0.833 7. MobileNet-V3 model = 0.819	1. ResNet18model = 0.802 2. ResNet-34 model = 0.810 3. ResNet-50 model = 0.857 4. DenseNet-121 model = 0.823 5. DenseNet-201 model = 0.823 6. MobileNet-V2 model = 0.823 7. MobileNet-V3 model = 0.823	NP	NP	1. ResNet18model = 0.806 2. ResNet-34 model = 0.822 3. ResNet-50 model = 0.836 4. DenseNet-121 model = 0.818 5. DenseNet-201 model = 0.816 6. MobileNet-V2 model = 0.824 7. MobileNet-V3 model = 0.799	None	None
Rekawek et al. [32]	A web-based implant failure and peri-implantitis prediction model	1. LR model of PI = 0.640 2. LR + bagging model of PI = 0.628 3. Random Forest model of PI = 0.746 4. SVM model of PI = 0.624 5. SVM + bagging model of PI = 0.606 6. XGB model of PI = 0.745 7. LR model of IF = 0.640 8. LR + bagging model of IF = 0.661 9. Random Forest model of IF = 0.735 10. SVM model of FI = 0.715 11. SVM + bagging model of Fi = 0.690 12. XGB model of FI = 0.717	1. LR model of PI = 0.341 2. LR + bagging model of PI = 0.317 3. Random Forest model of PI = 0.512 4. SVM model of PI = 0.268 5. SVM + bagging model of PI = 0.220 6. XGB model of PI = 0.573 7. LR model of IF = 0.462 8. LR + bagging model of IF = 0.462 9. Random Forest model of IF = 0.558 10. SVM model of FI = 0.481 11. SVM + bagging model of FI = 0.423 12. XGB model of FI = 0.558	NP	1. LR model of PI = 0.609 2. LR + bagging model of PI = 0.591 3. Random Forest model of PI = 0.875 4. SVM model of PI = 0.786 5. SVM + bagging model of PI = 0.9 6. XGB model of PI = 0.759 7. LR model of IF = 0.490 8. LR + bagging model of IF = 0.558 9. Random Forest model of IF = 0.707 10. SVM model of FI = 0.781 11. SVM + bagging model of FI = 0.786 12. XGB model of FI = 0.630	NP	1. LR model of PI = 0.810 2. LR + bagging model of PI = 0.804 3. Random Forest model of PI = 0.878 4. SVM model of PI = 0.825 5. SVM + bagging model of PI = 0.825 6. XGB model of PI = 0.862 7. LR model of IF = 0.720 8. LR + bagging model of IF = 0.751 9. Random Forest model of IF = 0.815 10. SVM model of FI = 0.820 11. SVM + bagging model of FI = 0.828 12. XGB model of FI = 0.788	None	None

*NP* not provided, *95%CI* Confidence interval of 95%, *AUROC* Area Under the Receiver Operating Characteristic curve, *Sen* Sensitivity, *Spec* Specificity, *PV* + Positive predictive value, *PV − *Negative predictive value, *Accu.* Accuracy, *PI* peri-implantitis, *IF* implant failure

Of the 35 models reporting AUROC values, 12 showed excellent discrimination (0.9 ≤ AUC), four showed very good discrimination capacity (0.8 ≤ AUC < 0.9), ten showing good discrimination potential (0.7 ≤ AUC < 0.8), with nine showing moderate discrimination potential (0.6 ≤ AUC < 0.7), irrespective of outcome definition. Of the 16 excellent or very good models, four were developed by the statistical modelling method, three were developed by the machine learning method, eight were developed by deep learning method and one was developed by esemble method (LR combined with AI method). Artificial intelligence prediction modeling is to use computer algorithms to discern patterns from data and predict clinical outcomes [41]. Within artificial intelligence, machine learning is a specific subset that enables machines to learn from data autonomously while deep learning is a specialized approach within machine learning that uses layered neural networks to process and analyze complex patterns in data [41]. We observed that the most frequently used factors in the 16 excellent or very good models were radiographic feature and implant position. Additionally, Four studies used more than two methods for model developement, and among them, the discrimination ability of LR models is on par with ML models in general.

Only three studies tested the external validation of the models, with two study conducting external validation by calculating the c-statistics and the other performing calculating the root-mean squard error.

### Risk of bias assessment

**Fig. 3:**
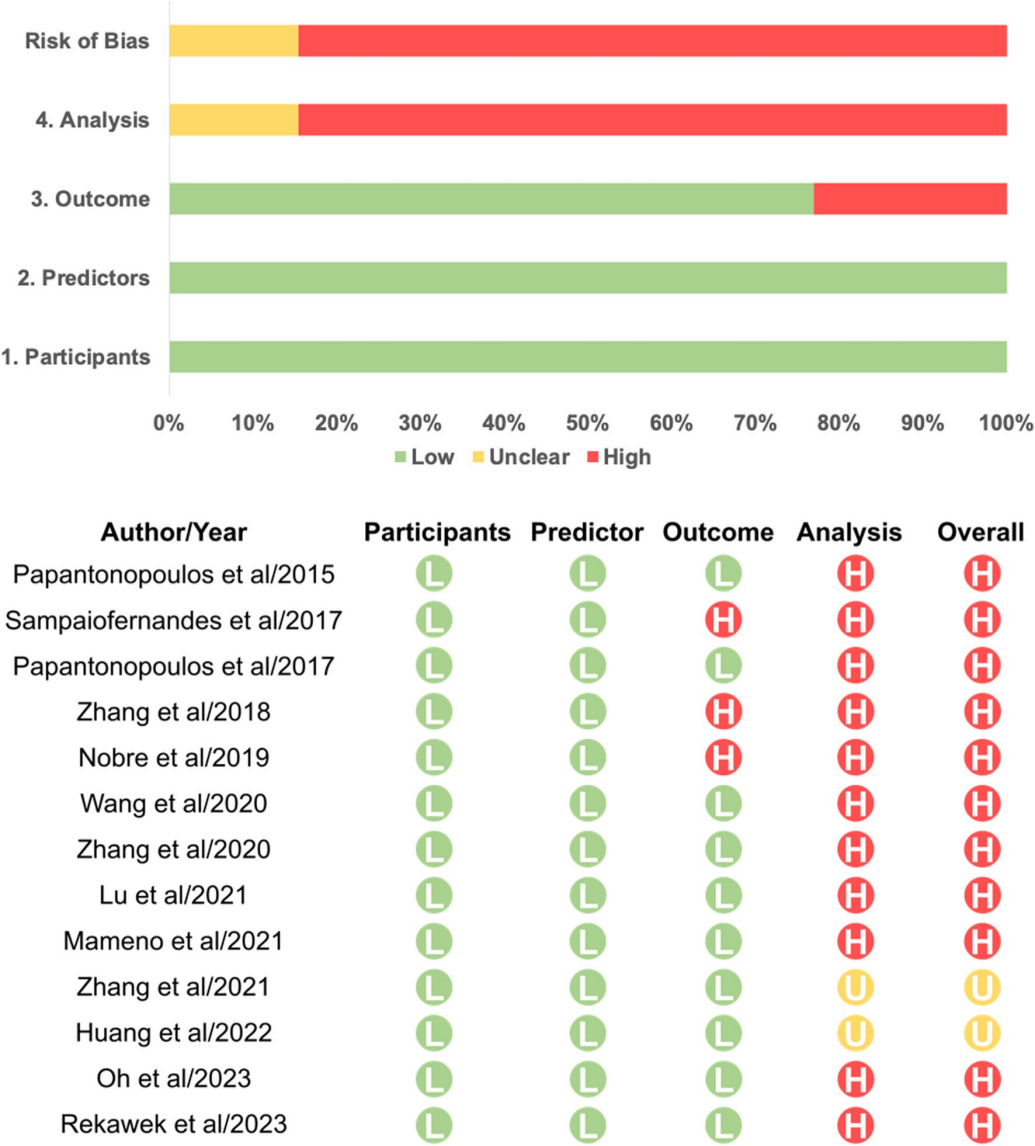
PROBAST (Prediction model Risk Of Bias ASsessment Tool) Quality Analysis Results of 10 Studies. Domain-level summary of risk of bias for all developed prediction models within the reviewed literature. **A** Overall rate of risk of bias (ROB) based on PROBAST: low, green; high, red; unclear, yellow. **B** ROB in each study. *L* low, *H* high, *U* unclear

Figure 3 shows the risk of bias in prediction modeling studies. None of the selected studies exhibited participant-related or predictor-related bias, while outcome-related bias was present in three studies. However, all studies suffered from analysis-related bias. No studies were excluded because of quality or bias. PROBAST risk of bias assessment in the 14 modeling prediction study, answers for each signalling question and reasons for being answered “N/PN (No/Probably No)” for signalling question in PROBAST for 14 studies were shown in supplementary file (Table S8–10). Detailed criteria of the PROBAST checklist are provided as well (Table S11).

### Transparent reporting assessment

**Fig. 4:**
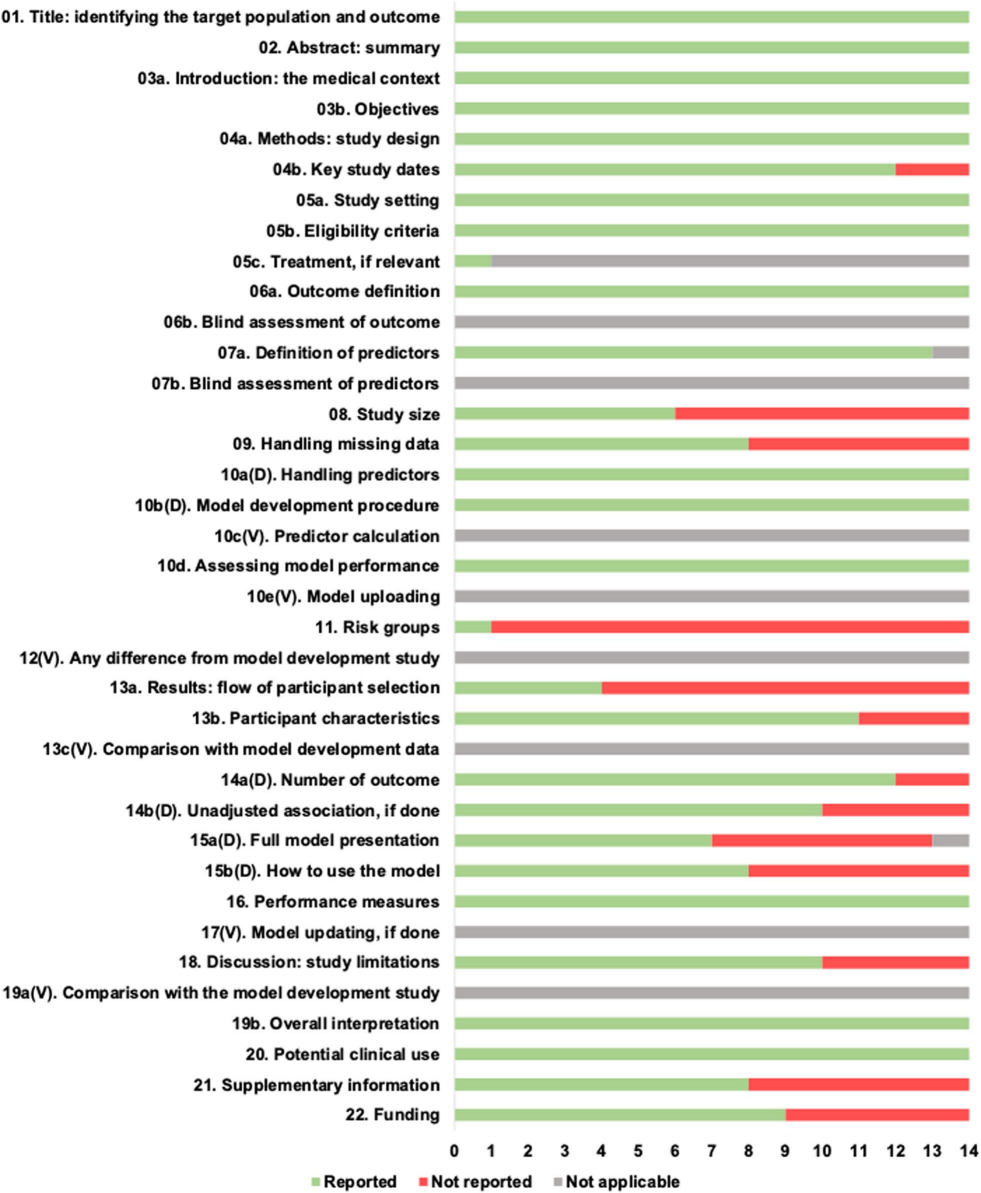
TRIPOD (Transparent Reporting of a Multivariable Prediction Model for Individual Prognosis or Diagnosis) Checklist. Numbers of development studies that reported each TRIPOD item. *D* development study only, *V* validation study only

Figure 4 displays the TRIPOD data for the 14 development studies, with a score range of 18‒27 (out of 31). All the studies met >69% of the items in the TRIPOD checklist. None of the studies were published before the TRIPOD’s publication on 7 January 2015. The most frequently reported items included the title (item 1), abstract (item 2) and introduction (item 3) for all the studies. Over 40% of the studies didn’t report missing data handling (item 9), full model presentation (item 15a), how to use the model (item 15b) and supplementary information. Important elements such as how they arrived at the sample size (item 8) and flow of participents selection (item 13a) were not reported in half of the studies.

### Feature importance

Most statistical models utilize predictors on disparate scales, obscuring the relative importance of each factor. Among the reviewed studies, only two—Lu’s and Wang’s—used predictors on the same scale [18, 19]. Both focused on genera as predictors. Wang’s study allows for an evaluation of relative importance based on the coefficients’ absolute values, with Caulobacter genera showing the highest impact [19]. However, Lu’s study does not detail the significance of individual predictors [18].

In AI models, feature importance was measured by different methods. In the study by Papantonopoulos [35], principal component analysis (PCA) is used to screen variables, where the eigenvalue can be used to measure the relative feature importance of the variables. The six parameters that showed eigenvalue ≥1 were in order of significance: number of remaining teeth, full-mouth plaque scores, implant surface, periodontitis severity, age and diabetes. In Zhang’s study [17], the variable importance plot of random forest model was presented by calculating the Gini index to present relative feature importance in which trabecular microarchitecture variables showed great importance. Mean values of feature importance in the random forest prediction model were presented in Mameno’s study [31]. The features importance was in order of significance: functional time, plaque control record, keratinized mucosa width, age, number of occlusal supports, smoking, brand, implant position, sex, history of periodontitis and fixation method. In two studies [36, 40] established deep learning-based predict model using radiography images, the feature importance could be estimated according to saliency map in which the proximity of the pixels in the image to the red hue on the color spectrum, which represents “high attention,” correlates with a more significant impact on the predictive outcomes of the model. In the study of Huang [36], cancellous alveolar bone with low relative density was highly correlated with implant loss in the saliency map. Rekawek’s study listed the feature importance for implant failure and peri-implantitis respectively [32]. The five most important characteristics of implant failure with were amount of local anesthetic, implant diameter, preoperative antibiotics, implant length, infrequent hygiene visits and the five most important features of peri-implantitis were implant diameter, implant length, preoperative antibiotics, infrequent hygiene visits, not diabetic. Studies by Ha [39] didn’t provided information about relative feature importance.

#### Overfitting

The fitness of models presented in Table S9–10 in the supplement data. The study by Papantonopoulos undertook external validation on a fresh dataset and disclosed a root-mean-squared error (RMSE) of 0.141 contrasted with the RMSE of 0.017 from the preceding study, this discrepancy implies a possible overfitting issue in the original research [34, 35]. Nobre’s investigation utilized the Hosmer and Lemeshow test for goodness-of-fit, yielding a *p*-value of 0.211, which indicates a good model fit [37]. Additionally, the external validation c-statistics of 0.709, albeit slightly lower than the derived set, remains within an acceptable threshold, signifying that the model retains predictive potency with new data samples. In the works of Zhang and Huang, calibration plots were executed, demonstrating a close alignment between the predicted and ideal curves across both studies [33, 36]. Specifically, in Zhang’s research, the C-index for the training and validation sets were 0.867 and 0.822, respectively, reinforcing the model’s satisfactory fitness [36].

Moreover, various AI model studies have implemented strategies to mitigate overfitting: Huang [36], Ha [39], Mameno [31], and Rekawek [32] employed cross-validation as a mechanism to combat overfitting. Huang further elucidated the process by presenting learning curves derived from fivefold cross-validation [36]. Oh’s study introduced on-the-fly data augmentation techniques during the training phase, utilizing random rotations to prevent overfitting [40]. However, Sampaio Fernandes [38], Zhang [17], Wang [19], and Lu [18], did not explicitly mention addressing the issue of overfitting in their research.

#### Dataset size relative to variable count

Among the studies encompassed in our review, three studies by Wang [19], Zhang [17], and Lu [18] respectively, have been identified with an events-per-variable ratios (EPV) less than 10, as delineated in Table S10.

## Discussion

The present systematic review summarized and appraised the prediction models for the complications and survival rates of dental implants. Firstly, predictors including implant length, implant position, aging, and history of periodontitis were consistently used in multiple prediction models. Second, there was limitation in the selection of predictors and inconsistency of diagnostic criteria of peri-implantitis. Third, the predictive ability of ML and LR models were similar. Fourth, reporting transparency, methodological quality, and external validity of prediction models were inadequate.

Several studies have reviewed the clinical prediction models in dentistry [42–44]. Consistent with our findings, a study that reviewed deep learning models in oral implantology concluded that many studies exhibited a high risk of bias [43]. High risk of bias might endanger the validity of findings, leading to potentially incorrect conclusions. It can lead decision-makers to choose ineffective treatments, wasting resources and potentially harming patients. Rigorous methods are essential to minimize bias, and caution is needed when interpreting results of clinical model research, especially when they guide clinical practice and policy. Another study reviewed the prediction models in periodontology [42]. Despite a general risk of bias and low transparency, prediction models in periodontology exhibited better methodological quality than those in implantology because more models in periodontology were tested concerning the calibration and discrimination performance than those in implantology. This may be because predictive models were first applied in periodontics and then extended to the implant field. Another meta-analysis showed that external validation studies of caries risk assessment models revealed that these models’ average predictive performance seems acceptable [44]. Prediction models in the dental caries assessment are more externally validated than those in implantology, possibly due to the higher incidence and easier diagnostic criteria for dental caries. It is essential to address these issues and conduct rigorous external validation to improve the reliability and generalizability of prediction models in dentistry.

In this systematic review, the most commonly used predictors were implant length and implant positions, followed by age and history of periodontitis. A systematic review and meta-analysis by Maha Abdel-Halim et al. examined the failure rates of short implants (<10 mm) and long implants (≥10 mm). The study results showed that the risk of failure for short implants was 2.5 times higher than for long implants, suggesting that implant length is a factor affecting implant failure [45]. Another study, using a multivariate marginal Cox analysis, provided suggestive evidence that implant length (<10 mm) was associated with an increased risk of implant failure [46]. The implant position factors mentioned here include the site of placement in jaw (anterior/posterior), the jaw (maxilla/mandible), and the three-dimensional position of the implant. Even when significant association with position is identified, from the articles we included, both anterior and posterior dental regions might be risk factors for implant survival or complications. The anterior implants’ susceptibility to complications could be attributed to its typically attenuated cortical bone compared to posterior implants [47], whereas the posterior area’s propensity for complications or implant loss may stem from its role in withstanding the primary occlusal force [48]. Furthermore, the anterior regions of both the maxilla and mandible typically possess a denser cortical layer compared to the posterior maxillary area. This dense bone is conducive to achieving primary implant stability, but also lead to the increased potential for thermal damage during the preparation of implant sites in dense bone can result in soft tissue encapsulation instead of the desired osseointegration [49]. The contrasting effects of dense bone might account for these divergent outcomes. But it is clear that the malpositioning of dental implants has been identified as an important factor in the incidence of implant-related complications and their survival rates. Specifically, inadequate inter-implant or implant-tooth distances may precipitate marginal bone loss, adversely affecting the osseointegration and prognostic stability of the implant. Concurrently, even mild implant malpositioning may give rise to inconsistency between the implant axis and occlusal forces, leading to complications [48].

In addition, older adults are more susceptible to periodontal and peri-implant diseases, which can be attributed to poor oral hygiene due to decreased dexterity and vision [50]. According to a retrospective cohort study, partially edentulous patients with a history of severe periodontitis were more prone to developing peri-implantitis [51]. Periodontitis grade of peri-implantitis patients was correlated to the severity of peri-implantitis and the occurrence of implant failure [52]. Furthermore, radiographic feature and implant position were the two most frequently used predictors among the excellent or very good models. These two predictors might improve the prediction ability of models.

Predictor selection is a critical aspect of model construction in prediction studies. First, it should be emphasized that causal factors clearly affect the outcomes, while predictors selected in prediction models do not necessarily need to have such causal effects [53]. Although using causal factors as predictors can enhance model portability across different populations and improve model reliability and acceptance, predictor selection should primarily focus on enhancing model differentiation and calibration [54]. Second, the statistical method for predictor selection is also important. In the included studies, the selection of variables depended on univariable analysis, possibly leading to incorrect predictor selection since the predictors are selected based on their statistical significance as a single predictor instead of being in context with other predictors, thus resulting in the omission of variables from the model [12, 55]. Third, it is essential to make the models applicable to daily practice. Ideal predictors should be inexpensive, easy to obtain, and accurate [12]. We noted that some models use a combination of clinical, demographic, and molecular measures to predict implant retention and complications. Although this seems to improve the performance of the models, some predictors (i.e. microbial predictors, molecular measures) are cumbersome to measure, which often makes the model less useful in the clinic.

Four studies predicted the occurrence of peri-implantitis as the most common outcome among the 14 prediction modeling studies. Regarding peri-implantitis prediction, studies have used different definitions, leading to inconsistent models. The widely acknowledged diagnostic criteria for peri-implantitis, formulated based on the 2017 consensus workshop (Table S12), require (1) the presence of bleeding and/or suppuration on gentle probing, (2) increased probing depth compared to previous examinations, and (3) the presence of bone loss beyond crestal bone level changes resulting from initial bone remodeling.

All studies were published after the consensus meeting. In 3 of those studies, the definition was made according to the consensus report of workgroup 4 of the 2017 world workshop [15, 30–32], highly consistent with the norms in this aspect. The definition of peri-implantitis in two models from Zhang’s study followed the consensus [15, 30], which included the presence of bleeding and/or suppuration on probing, increased probing depth compared to previous examinations, and bone loss beyond crestal bone level changes after initial bone remodeling. In Mameno’s study with three models, peri-implantitis was defined as the presence of bleeding on probing and/or suppuration in the follow-up period and the presence of >1 mm of bone resorption from the baseline measurement [31]. In Rekawek’s study, peri-implantitis was defined as radiographic evidence of changes in the crestal bone level and clinical evidence of bleeding on probing, with or without suppuration [32]. However, the definition of peri-implantitis in Zhang’s study [33] included bleeding/ suppuration on probing, probing depth ≥5 mm, and radiographic marginal bone loss ≥2 mm while probing depths of ≥6 mm were suggested in the consensus. The overly broad diagnostic criteria in this study could result in false positives. It is suggested that future studies strictly adhere to the diagnostic criteria in 2017 World Workshop on the Classification of Periodontal and Peri-Implant Diseases and Conditions (Table S12) to increase comparability between studies and promote the generalizability of the models. It bears emphasis that the diagnostic criteria encompass two sets: one with and one without baseline data. While the absence of baseline data is a common clinical scenario, literature suggests that its presence enhances diagnostic precision [56]. In its absence, the use of secondary diagnostic criteria may demonstrate diminished sensitivity, risking the omission of early or subtle peri-implantitis cases.

In terms of the proportion of models with excellent and very good AUROC, the model constructed by deep learning method has the best discrimination. Machine learning models (except for deep learning models) and logistic regression models were similar in performance in terms of the AUROC value. Four studies used multiple methods to develop models, demonstrating varying discrimination levels for different models. Zhang et al. showed that the support vector machine (SVM), artificial neural network (ANN), and logistic regression models exhibited a high sensitivity of 91.67% [17]. In Mameno’s study, the support vector machine model outperformed the logistic regression and random forest (RF) models [31]. Huang et al. [36] demonstrated that the integrated model they developed combining the logistic regression and convolutional neural network models showed better discrimination than both of them separately. In Rekawek’s study, LR and SVM had similar discrimination while RF had the best performance [32]. These findings are consistent with previous studies that have not shown a performance benefit of ML models over LR models for clinical prediction [57]. When the data meet the criteria for using logistic regression, it may be preferable as it provides more interpretable and explanatory results than ML methods [58]. Therefore, clinicians should consider the method’s suitability rather than inappropriately favor advanced and complex techniques. Though the deep learning model outperforms the statistical model in terms of the amount of excellent and very good AUROC models, it is more susceptible to overfitting. Additionally, these studies lacked external validation, making it challenging for us to determine superiority. Moreover, the burgeoning field of applied machine learning in predictive modeling introduces layers of complexity, heightened by their variable quality that sometimes makes the application of these models remain challenging [59]: Firstly, it brings algorithmic complexity. Complex machine learning algorithms demand meticulous tuning and can overfit, performing well on training data but poorly on new instances. Secondly, AI models have data reliance, posing challenges in domains with scant data resources [60]. In addition, many AI models, particularly deep learning ones, has interpretability gaps, complicating the task of explaining their decisions [61].

The TRIPOD checklist revealed low transparency in reporting the prediction models. Over 40% of the studies didn’t report missing data handling (item 9), full model presentation (item 15a), how to use the model (item 15b) and supplementary information. The lack of a full model presentation might prompt other researchers to develop a new model instead of conducting external validation research, resulting in many prediction model development publications failing to be implemented in the clinic. Important elements such as how they arrived at the sample size (item 8) and flow of participents selection (item 13a) were not reported in half of the studies. Selective reporting of models might cause a lack of authenticity and insufficient evidence, resulting in over-optimism of the performance of these models [20].

The poor quality of the model is mainly attributed to the following three aspects: the method of dealing with missing data, lack of proper calibration assessment and the lack of external validation. Of the eight studies that reported how to deal with missing data, one study used K-nearest-neighbors imputation methods, while the remaining seven studies reported excluding subjects with missing data as the method for handling missing data. Excluding subjects with missing data can be reasonable when the number of missing cases is small. However, it leads to a significant reduction in the sample size and potential bias when many variables have missing data. Then, in the only three studies that conducted calibration assessment, only two models presented calibration plot, while the other reporting only the Hosmer–Lemeshow test with no calibration plot or a table. This makes it impossible to provide effective information for evaluating the accuracy of the predicted risks in comparing the predicted versus observed outcome frequencies. Moreover, only three model conducted external validation of all the 14 studies. External validation is crucial for evaluating the model’s generalization ability and ensuring its good performance on new data, thereby enhancing the accuracy and reliability of the prediction models [62]. A key factor hindering the clinical implementation of the prediction models is that most models have a good prediction ability in the developing set but are poor in the external validation set. Therefore, it is necessary to conduct external validation in our clinical modeling process.

For statistic model studies, a larger absolute value of a coefficient typically indicates a more important feature, assuming all features are on the same scale. However, most of the statistic models use different types of predictors which make it difficult to determine the relative feature importance. As for the articles reported with this relative feature importance, the results are too heterogeneous to be compared. Calculating the feature importance plays a pivotal role in the realm of machine learning and statistical modeling. It aids in the strategic selection of features, enabling the identification of the most critical elements that can streamline the model and enhance its clarity and workability [63]. Additionally, it enriches the model’s interpretability, offering a tangible method to elucidate the underpinnings of its predictive mechanisms [61].

The included clinical prediction models necessitate evaluation against overfitting, typically through external validation (calculating RMSE, c-statics) on a distinct dataset or by employing cross-validation techniques. There are also some other methods like utilizing the Hosmer and Lemeshow test for goodness-of-fit, presenting calibration plots, employing on-the-fly data augmentation techniques as mentioned in the included studies. However, about one third of the included studies failed to address overfitting which might result in poor generalization, decreased predictive performance and increased complexity, especially in AI models.

Three of the included studies identified with an EPV less than 10. Low EPV indicate that the model is at risk of overfitting, or that more samples are needed to ensure the stability and generalization of the results. Some method can be utilized to address this issue. Penalty regression (such as ridge regression [64], lasso regression [65] and elastic net [66], etc.) is a machine learning algorithm for regression tasks where the goal is to prevent overfitting by adding a penalty term. Each of these methods has its own advantages and shortcomings. Ridge Regression reduces coefficient sizes but keeps all variables, which can’t fully prevent overfitting as effectively as methods that can eliminate variables and may still include irrelevant variables [64]. Lasso Regression trims coefficients to zero for variable selection, enhancing model simplicity and interpretability, but risks excluding crucial predictors [65]. Elastic Net strikes a balance by blending both penalties of Lasso and Ridge regression, yet it demands meticulous tuning of its parameters, posing a computational challenge [66].

However, EPV is a guiding principle rather than a hard and fast rule. It is used as an auxiliary tool for sample size calculation to ensure model stability and avoid overfitting. Researchers should consider all relevant factors, such as study design, effect size, and statistical power, etc.

The duration of follow-up across the reviewed studies spans from a minimum of six months to a maximum exceeding five years. Within these studies, four out of the 14 studies have a follow-up duration of one year or less. While this timeframe is adequate for monitoring soft tissue alterations, such as BOP and SOP, it falls somewhat short for comprehensively assessing bone tissue remodeling after implant surgery. Consequently, due to the fact that bone tissue remodeling requires a more extended period and significant bone loss can still occur after the implant has been in function for over a year [67], a one-year follow-up period may be deemed relatively brief for studies aimed at evaluating bone tissue such as marginal bone loss. Sampaio-Fernandes et al.’s 6-month follow-up was adequate for tracking mobility, pain, peri-implant mucositis but insufficient for peri-implantitis assessment [38]. Also, the one-year follow-ups in the Nobre et al. and Zhang et al. trials were too short for comprehensive peri-implantitis evaluation [37, 47]. Ha et al.’s one-year follow-up, focusing on the outcome measures were implant survival and complications that did not involve bone tissue assessment, was reasonable for the study’s objectives [68].

It should be noted that the article’s approach to resolving inconsistencies in measurement or operations varies. A case in point is the variable of multiple physicians, which is recognized as a potential factor affecting the outcome but is not uniformly addressed. In the two studies conducted by Papantonopoulos et al. [34, 69], the consistency of clinical and radiographic measurements was maintained by assigning a single operator to perform these assessments. Similarly, Ha [68], Lu [18], Wang [19], and Oh [40] controlled for variability by having the operations conducted by one operator following a standardized protocol. In contrast, Zhang et al. [15], who used three experienced operators for implant placements, did not report measures for inter-operator reliability. The other studies lacked details on data collection reliability and did not calculate stability or consistency measures, nor did they adjust for inter-rater reliability, potentially introducing bias when multiple physicians were involved.

Several findings have been listed to improve the use and performance of clinical prediction models. First, improving the model’s performance and simplicity should be a priority when selecting the predictors rather than giving a causal interpretation of predictors from a model. An ideal prediction model should exhibit a balance between predictability and simplicity. Second, a consistent definition of peri-implantitis can enhance the comparability between studies and the generalization of models. The diagnostic criteria in the consensus report of the 2017 world workshop are highly recommended for further research. Third, when selecting the model development method, it is crucial to consider the data and clinical application rather than rely on advanced methods inappropriately. Fourth, clinical prediction modeling studies should conform to the standards outlined in the TRIPOD statement to ensure reporting completeness and transparency. Fifth, proper missing data analysis, calibration and external validation should be conducted to improve the quality of the model. The PROBAST checklist can be utilized to mitigate the risk of bias.

This systematic review provided suggestions for future studies on models in implantology. Snowball strategy showed that only one literature in references was found, indicating the completeness of the search strategy. This study had some limitations. First, research results of the grey literature were not included due to its unavailability. Additionally, studies without accurate values, such as AUROC, sensitivity, specificity, PV+, PV−, and accuracy, were excluded to comprehensively analyze the models. The limitations of this review study in terms literature sources should be mentioned. Furthermore, despite many of them being retrospective studies, there is also heterogeneity of study designs, population, outcome definitions, developing methods, etc. The heterogeneity and a limited number of high-quality studies restrict the feasibility of performing a meta-analysis. Lastly, to ideally assess predictive factors, prospective longitudinal data is to be utilized. We included 12 retrospective studies study considering the few amounts of the included studies. Future studies need to use more prospective cohort studies to build model.

## Conclusion

This review assessed the prediction models for dental implant complications and survival rates, and found a common shortage of methodological quality in the prediction models. Although the models showed good predictive capacity, their external validity was inadequate, only three study had external validation. Future research should aim to increase the generalization of the models, improve the methodological quality of models, and improve the repoting transparency of the studies. Particular focus is also needed on the clinical utility and applicabilty of these prediction models. Two predictors, i.e., radiographic feature and implant position, can potentially enhance the predictive ability of models predicting the complication and survival rates of dental implants. More research is needed to demonstrate its predictive performance in models.

## Supplementary Information

Additional file 1.

## Data Availability

No datasets were generated or analysed during the current study.

## Abbreviations

PROBAST: Prediction Model Risk of Bias Assessment Tool
TRIPOD: Transparent Reporting of a Multivariable Prediction Model for Individual Prognosis or Diagnosis
PRISMA: Preferred Reporting Items for Systematic Reviews and Meta-Analyses
PROSPERO: International prospective register of systematic reviews
AUROC: The area under the receiver operating characteristic curve
PV+: Positive predictive value
PV−: Negative predictive value
CHARMS: Critical Appraisal and Data Extraction for Systematic Reviews of Prediction Modelling Studies
PD: Probing depth
MBL: Marginal bone loss
LR: Logistic regression
ML: Machine learning
DL: Deep learning
AI: Artificial intelligent
PCA: Principal component analysis
RMSE: Root-mean-squared error
EPV: Events-per-variable ratios
SVM: Support vector machine
ANN: Artificial neural network
RF: Random forest
