# Supplementary material for: Prediction models for the complication incidence and survival rate of dental implants—a systematic review and critical appraisal
Source: Int J Implant Dent. 2025 Jan 23;11:5. doi: 10.1186/s40729-025-00590-1 (PMC11757661; doi:10.1186/s40729-025-00590-1)
Supplement: Supplementary file 1 — Additional file 1. [file 40729_2025_590_MOESM1_ESM.docx]

**SUPPLEMENTAL FILES**

**Title:** Prediction models for the complication incidence and survival rate of dental implants–A systematic review and critical appraisal

**Authors:** Yuanxi Zhu, Mi Du, Ping Li, Hongye Lu, An Li, Shulan Xu

Table of Contents

[Table S1. PRISMA checklist 2](#_Toc180616929)

[Table S2. Complete query of the search strategy in Pubmed, Web of science and Embase database. 6](#_Toc180616930)

[Table S3. Excluded reasons for full text screening of studies via database search. 7](#_Toc180616931)

[Table S4. Excluded reasons for full text screening of studies via references. 10](#_Toc180616932)

[Table S6. Predictors identified not less than twice in the prediction models. 12](#_Toc180616933)

[Table S7. Candidate predictors for 14 modeling studies. 14](#_Toc180616934)

[Table S8. PROBAST risk of bias assessment in the 14 modeling prediction study. 17](#_Toc180616935)

[Table S9. Answers for each signalling question in PROBAST for 14 modeling prediction studies. 18](#_Toc180616936)

[Table S10. Reasons for being answered “N/PN (No/Probably No)” for signalling question in PROBAST for 14 studies. 20](#_Toc180616937)

[Table S11. Signalling questions for PROBAST. 21](#_Toc180616938)

[Table S12. Diagnosis of peri-implantits in the 2017 World Workshop on the Classification of Periodontal and Peri-Implant Diseases and Conditions. 23](#_Toc180616939)

# Table *S*1. PRISMA checklist

| **Section and Topic** | **Item #** | **Checklist item** | **Location where item is reported** |
| --- | --- | --- | --- |
| **TITLE** | | |  |
| Title | 1 | Identify the report as a systematic review. | Title (p1) |
| **ABSTRACT** | | |  |
| Abstract | 2 | See the PRISMA 2020 for Abstracts checklist. | Abstract (p2) |
| **INTRODUCTION** | | |  |
| Rationale | 3 | Describe the rationale for the review in the context of existing knowledge. | Intro (p3) |
| Objectives | 4 | Provide an explicit statement of the objective(s) or question(s) the review addresses. | Intro (p4) |
| **METHODS** | | |  |
| Eligibility criteria | 5 | Specify the inclusion and exclusion criteria for the review and how studies were grouped for the syntheses. | Method (p6) |
| Information sources | 6 | Specify all databases, registers, websites, organisations, reference lists and other sources searched or consulted to identify studies. Specify the date when each source was last searched or consulted. | Method (p5) |
| Search strategy | 7 | Present the full search strategies for all databases, registers and websites, including any filters and limits used. | Tab. S2 |
| Selection process | 8 | Specify the methods used to decide whether a study met the inclusion criteria of the review, including how many reviewers screened each record and each report retrieved, whether they worked independently, and if applicable, details of automation tools used in the process. | Method (p6-7) |
| Data collection process | 9 | Specify the methods used to collect data from reports, including how many reviewers collected data from each report, whether they worked independently, any processes for obtaining or confirming data from study investigators, and if applicable, details of automation tools used in the process. | Method (p7) |
| Data items | 10a | List and define all outcomes for which data were sought. Specify whether all results that were compatible with each outcome domain in each study were sought (e.g. for all measures, time points, analyses), and if not, the methods used to decide which results to collect. | Method (p7)  Tab. 1-3 |
|  | 10b | List and define all other variables for which data were sought (e.g. participant and intervention characteristics, funding sources). Describe any assumptions made about any missing or unclear information. | Method (p6)  Tab. 1-3 |
| Study risk of bias assessment | 11 | Specify the methods used to assess risk of bias in the included studies, including details of the tool(s) used, how many reviewers assessed each study and whether they worked independently, and if applicable, details of automation tools used in the process. | Method (p8) |
| Effect measures | 12 | Specify for each outcome the effect measure(s) (e.g. risk ratio, mean difference) used in the synthesis or presentation of results. | n/a |
| Synthesis methods | 13a | Describe the processes used to decide which studies were eligible for each synthesis (e.g. tabulating the study intervention characteristics and comparing against the planned groups for each synthesis (item #5)). | Method (p6) |
|  | 13b | Describe any methods required to prepare the data for presentation or synthesis, such as handling of missing summary statistics, or data conversions. | Method (p7) |
|  | 13c | Describe any methods used to tabulate or visually display results of individual studies and syntheses. | Tab. 1-3 |
|  | 13d | Describe any methods used to synthesize results and provide a rationale for the choice(s). If meta-analysis was performed, describe the model(s), method(s) to identify the presence and extent of statistical heterogeneity, and software package(s) used. | Method (p6) |
|  | 13e | Describe any methods used to explore possible causes of heterogeneity among study results (e.g. subgroup analysis, meta-regression). | n/a |
|  | 13f | Describe any sensitivity analyses conducted to assess robustness of the synthesized results. | n/a |
| Reporting bias assessment | 14 | Describe any methods used to assess risk of bias due to missing results in a synthesis (arising from reporting biases). | Method (p7) |
| Certainty assessment | 15 | Describe any methods used to assess certainty (or confidence) in the body of evidence for an outcome. | n/a |
| **RESULTS** | | |  |
| Study selection | 16a | Describe the results of the search and selection process, from the number of records identified in the search to the number of studies included in the review, ideally using a flow diagram. | Fig. 1 |
|  | 16b | Cite studies that might appear to meet the inclusion criteria, but which were excluded, and explain why they were excluded. | Fig. 1  Tab.S6-7 |
| Study characteristics | 17 | Cite each included study and present its characteristics. | Tab. 1-3 |
| Risk of bias in studies | 18 | Present assessments of risk of bias for each included study. | Fig. 3 |
| Results of individual studies | 19 | For all outcomes, present, for each study: (a) summary statistics for each group (where appropriate) and (b) an effect estimate and its precision (e.g. confidence/credible interval), ideally using structured tables or plots. | Tab. 3 |
| Results of syntheses | 20a | For each synthesis, briefly summarise the characteristics and risk of bias among contributing studies. | Fig. 3 |
|  | 20b | Present results of all statistical syntheses conducted. If meta-analysis was done, present for each the summary estimate and its precision (e.g. confidence/credible interval) and measures of statistical heterogeneity. If comparing groups, describe the direction of the effect. | n/a |
|  | 20c | Present results of all investigations of possible causes of heterogeneity among study results. | n/a |
|  | 20d | Present results of all sensitivity analyses conducted to assess the robustness of the synthesized results. | n/a |
| Reporting biases | 21 | Present assessments of risk of bias due to missing results (arising from reporting biases) for each synthesis assessed. | n/a |
| Certainty of evidence | 22 | Present assessments of certainty (or confidence) in the body of evidence for each outcome assessed. | n/a |
| **DISCUSSION** | | |  |
| Discussion | 23a | Provide a general interpretation of the results in the context of other evidence. | Discussion (p16) |
|  | 23b | Discuss any limitations of the evidence included in the review. | Discussion (p25) |
|  | 23c | Discuss any limitations of the review processes used. | Discussion (p25) |
|  | 23d | Discuss implications of the results for practice, policy, and future research. | Discussion (p24) |
| **OTHER INFORMATION** | | |  |
| Registration and protocol | 24a | Provide registration information for the review, including register name and registration number, or state that the review was not registered. | Method (p5) |
|  | 24b | Indicate where the review protocol can be accessed, or state that a protocol was not prepared. | Method (p5) |
|  | 24c | Describe and explain any amendments to information provided at registration or in the protocol. | n/a |
| Support | 25 | Describe sources of financial or non-financial support for the review, and the role of the funders or sponsors in the review. | Funding (p27) |
| Competing interests | 26 | Declare any competing interests of review authors. | Competing interests statement (p27) |
| Availability of data, code and other materials | 27 | Report which of the following are publicly available and where they can be found: template data collection forms; data extracted from included studies; data used for all analyses; analytic code; any other materials used in the review. | Table S1-S8 |

Checklist obtained from http://prisma-statement.org/PRISMAStatement/Checklist. n/a = not applicable. Page numbers based on accepted manuscript file.

# Table *S*2. Complete query of the search strategy in Pubmed, Web of science and Embase database.

| Database | Query |
| --- | --- |
| Pubmed | Search: "Dental implants"[MAJR] AND ("dental implant"[tiab] OR "dental implant"[tiab] OR implantology[tiab] OR "peri-implantitis"[mh] OR "peri-implantitis"[tiab] OR "implant loss"[tiab] OR "implant mobility"[tiab] OR "implant survival"[tiab] OR "implant failure"[tiab] ) AND ("forecasting"[mh] OR forecast*[tiab] OR predict*[tiab] OR "disease progression/epidemiology"[mh] OR "risk assessment/epidemiology"[mh] OR "risk assessment"[tiab] OR "risk factor*"[mh] OR "risk factors*"[tiab]) AND ("cohort studies"[mh] OR "cohort"[tiab] OR "longitudinal studies"[mh] OR "longitudinal"[tiab] OR "prospective studies"[mh] OR "prospective"[tiab] OR "follow-up studies"[mh] OR "follow up"[tiab] OR "retrospective studies"[mh] OR "retrospective"[tiab] ) |
| Web of science | TS= (“dental implant” OR “dental implant” OR "peri-implantitis" ) AND TS=(Predict* OR forecast* OR “risk assessment” OR “risk factor*”) AND TS= (“cohort study” OR “cohort studies” OR “longitudinal study” OR “longitudinal studies” OR “prospective study” OR “prospective studies” OR “retrospective study” OR “retrospective studies” OR “follow up” OR “follow-up”) AND |
| Embase | #1 AND #2 AND #3  #1: “dental implant”/exp OR “dental implant” OR “dental implant” OR “implantology” OR “implant loss”/exp OR “implant loss” OR “implant mobility” OR “peri-implantitis”/exp OR “peri-implantitis” OR “periimplantitis”/exp OR “periimplantitis” OR “peri-implant disease”  #2: “prediction” OR “forecasting” OR “predictive” OR “risk assessment” OR “risk factor”  #3: “longitudinal” OR “prospective” OR “retrospective” OR “follow up” OR “follow-up” |

# Table *S*3. Excluded reasons for full text screening of studies via database search.

| **Number** | **Author & Year** | **Title** | **Excluded reason** |
| --- | --- | --- | --- |
| 1 | Huang, H., 2017 | Multivariate linear regression analysis to identify general factors for quantitative predictions of implant stability quotient values | Wrong Outcome |
| 2 | Li, Y., 2020 | Correlation between the morphology of alveolar bone defect in the maxillary anterior region and the outcome of guided bone regeneration | Wrong language |
| 3 | Maruo, Katsuichiro, 2016 | A retrospective study to compare improvement of implant maintenance by Medical Treatment Model | Validation study |
| 4 | Testori, T., 2016 | A Retrospective Analysis of the Effectiveness of the Longevity Protocol for Assessing the Risk of Implant Failure | Validation study |
| 5 | De Araújo Nobre, M., 2019 | A peri-implant disease risk score for patients with dental implants: Validation and the influence of the interval between maintenance appointments | Validation study |
| 6 | De Ry, S. P., 2021 | Evaluation of the implant disease risk assessment (IDRA) tool: A retrospective study in patients with treated periodontitis and implant-supported fixed dental prostheses (FDPs) | Validation study |
| 7 | Sarbacher, Amelie, 2022 | Comparison of Two Risk Assessment Scores in Predicting Peri-Implantitis Occurrence during Implant Maintenance in Patients Treated for Periodontal Diseases: A Long-Term Retrospective Study | Validation study |
| 8 | Mo, J. J., 2022 | Long-term clinical outcomes of short implant (6mm) in relation to Implant Disease Risk Assessment (IDRA) | Validation study |
| 9 | Atieh, Momen A., 2014 | The prognostic accuracy of resonance frequency analysis in predicting failure risk of immediately restored implants | Single factor |
| 10 | Kim, S. J., 2015 | Resonance frequency analysis as a predictor of early implant failure in the partially edentulous posterior maxilla following immediate nonfunctional loading or delayed loading with single unit restorations | Single factor |
| 11 | Wentaschek, Stefan, 2015 | Sensitivity and Specificity of Stability Criteria for Immediately Loaded Splinted Maxillary Implants | Single factor |
| 12 | Baltayan, S., 2016 | The predictive value of resonance frequency analysis measurements in the surgical placement and loading of endosseous implants | Single factor |
| 13 | Lupi, S. M., 2019 | Detection of peri-implant inflammation by the use of a matrix metalloproteinase-8 chair-side test | Single factor |
| 14 | Jacobi-Gresser, E., 2013 | Genetic and immunological markers predict titanium implant failure: a retrospective study | Risk association study |
| 15 | Rodriguez, M. V., 2022 | Is the degree of physiological bone remodeling a predictive factor for peri-implantitis? | Wrong exposure |
| 16 | Chuang, S. K., 2002 | Predicting dental implant survival by use of the marginal approach of the semi-parametric survival methods for clustered observations | No accurate value |
| 17 | Chuang, S. K., 2006 | Predicting clustered dental implant survival using frailty methods | No accurate value |
| 18 | Merheb, J., 2015 | Prediction of implant loss and marginal bone loss by analysis of dental panoramic radiographs | No accurate value |
| 19 | Canullo, Luigi y, 2017 | Association Between Clinical and Microbiologic Cluster Profiles and Peri-implantitis | No accurate value |
| 20 | Mazel, A., 2019 | Peri-implantitis risk factors: A prospective evaluation | No accurate value |
| 21 | Jeong, I. C., 2019 | Implant Failure Prediction Using Discriminant Analysis | No accurate value |
| 22 | Atieh, M. A., 2019 | Predicting peri-implant disease: Chi-square automatic interaction detection (CHAID) decision tree analysis of risk indicators | No accurate value |
| 23 | Wieczorek, K., 2019 | Predictive values of resonance frequency analysis as a diagnostic tool in palatal implant loss | No accurate value |
| 24 | Yi, Y., 2020 | Association of prosthetic features and peri-implantitis: A cross-sectional study | No accurate value |
| 25 | Radaelli, M. T. B., 2020 | Early-predictors of marginal bone loss around morse taper connection implants loaded with single crowns: A prospective longitudinal study | No accurate value |
| 26 | Feher, B., 2020 | An advanced prediction model for postoperative complications and early implant failure | No accurate value |
| 27 | de Araujo Nobre, 2015 | Risk factors of peri-implant pathology | Wrong study design |

# Table *S*4. Excluded reasons for full text screening of studies via references.

| **Number** | **Author & Year** | **Title** | **Excluded reason** |
| --- | --- | --- | --- |
| 1 | Luterbacher, S.2000 | Diagnostic characteristics of clinical and microbiological tests for monitoring periodontal and peri-implant mucosal tissue conditions during supportive periodontal therapy (SPT) | No accurate value |
| 2 | Chuang, S. K.2001 | Kaplan-Meier analysis of dental implant survival: a strategy for estimating survival with clustered observations | No accurate value |
| 3 | Okayasu, Kozue2011 | Decision Tree for the Management of Periimplant Diseases | No accurate value |
| 4 | Braga AC2012 | Decision Model to Predict the Implant Success | No peer reviewed |
| 5 | Ha, Seung-Ryong2018 | A pilot study using machine learning methods about factors influencing prognosis of dental implants | No accurate value |

**Table *S*5. Predictors identified not less than twice in the prediction modeling studies.**

| Risk predictors | Frequency identified, n (%)* (N = 15) |
| --- | --- |
| Implant position | **6 (11.1)** |
| Implant length | **6 (11.1)** |
| Age | **5 (9.3)** |
| History of periodontitis | **5 (9.3)** |
| Smoking | **4 (7.4)** |
| Gender | **4 (7.4)** |
| Implant diameter | **4 (7.4)** |
| Blood glucose | **4 (7.4)** |
| Microbial predictor | **3 (5.6)** |
| Radiograpic features | **3 (5.6)** |
| Fixation method | **2 (3.7)** |
| Plaque control | **2 (3.7)** |
| Probing depth | **2 (3.7)** |
| Functional time | **2 (3.7)** |
| Number of remaining teeth | **2 (3.7)** |

***: Analys unit is study**

# Table *S*6. Predictors identified not less than twice in the prediction models.

| Risk predictors | Frequency identified, (N = 32) |
| --- | --- |
| Implant length | 20 |
| Implant diameter | 18 |
| Implant position | 16 |
| Infrequent hygiene visits | 12 |
| Preoperative antibiotics | 12 |
| Age | 11 |
| Gender | 10 |
| Periodontitis | 9 |
| Blood glucose | 9 |
| Smoking | 7 |
| Retention method | 7 |
| Radiographic DL predictors | 7 |
| Amount of local anesthetic | 6 |
| Functional time of the implant | 4 |
| Structure model index (SMI) | 4 |
| Bone surface volume ratio (BS/BV) | 4 |
| Percent bone volume (BV/TV) | 4 |
| Mesio-distal position for restoration | 4 |
| Cortical bone thickness | 4 |
| Trabecular pattern factor (Tb.Pf) | 4 |
| Number of occlusal supports | 3 |
| PCR | 3 |
| Number of remaining teeth | 3 |
| Implant brand | 3 |
| Number of cigarettes smoked | 3 |
| KMW | 3 |
| Tannerella | 2 |
| bucco-lingual angulation | 2 |
| implant torque | 2 |
| Immediate implantation | 2 |
| Aggregatibacter | 2 |
| Bone graft | 2 |

# Table *S*7. Candidate predictors for 14 modeling studies.

| Study | | Candidate predictors |
| --- | --- | --- |
| Author/Year | Model |  |
| Papantonopoulos et al/2015 | Predictive model for peri-implantitis | Age, gender, smoking, diabetes, other medical conditions, years of implant function, compliance with recall, number of teeth, periodontitis severity level, plaque level, missing molars occlusion, cantilever in prostheses and number of implants. |
| Sampaiofernandes et al/2017(Sampaio Fernandes et al., 2017) | Prediction model for biological complications of implant overdentures | Medical treatment, Surgery, Implant technique, Postsurgical, Average plaque modified index, Mean probing depth (PD), Mean attached gingiva, Metal exposure, IL1A_allele2, IL1B_allele2, IL-1 Genotype, A. actinomycetemcomitans, B. forsythus, F. nucleatum, P. gingivalis, Maxillary edentulousness, Gingiva hypertrophy |
| Papantonopoulos et al/2017.7 | Prediction model of individual implant bone | Age of the patient having the implants at the time of the examination, gender, FMPS, compliance rate with recall schedule, number of remaining teeth, number of implants inserted in a patient, number of years of implant function, smoking, the presence of self-reported diabetes, other medical conditions, periodontitis severity by % of teeth with ≥50% of bone loss on radiographs, the presence of cantilevers in the prosthetic design, type of overlying prosthesis (single, multi-unit or overdenture), missing molar occlusion, implant surface, implant diameter and jaw bone site in which an implant was inserted. |
| Zhang et al/2018(Zhang et al., 2018) | Nomogram prediction model of peri-implantitis | Length of implant, Diameter of implant, Jaw, Position, Prosthetic type, Bone regeneration, Sinus lift, Bone regeneration (split out the sinus lift), Mean PDi (mm), Max-PDi (mm), Mean Bii, △MBL-mesial (mm/year), △MBL-distal (mm/year), Mean PD T1 (mm), PD T1 ≥ 6 mm (%), Mean BI, T1 (mm), FMBS T1, BI T1 ≥ 3 (%), Mean PD T2 (mm), PD T2 ≥ 6 mm (%), Mean BI T1 (mm), FMBS T2, BI T2 (≥3%) |
| Ha et al/ 2018.12(有待引用) | Prognosis model of dental  implants | Immediate implantation. Implant insertion depth, Bucco-lingual angulation, Mesio-distal position for restoration, Implant length, Crown-root ratio, Site of placement, Site of placement in jaw, Posterior site of placement, Bone sufficiency, Soft tissue problem, Bone graft, Bone graft resorption. |
| de Araújo Nobre et al/2019(Nobre et al., 2019) | A prognostic model for ailing and failing implants due to peri-implant disease | Number of patients, Average age, Gender, Implant status (Survival, Failure), History of Periodontitis, Systemic comorbidities, Smoking, Proximity of implants/teeth, Type of implant surface, Implant position per arch, Implant position in the arch, Time of follow-up at diagnosis, Implant length, Abutment height, Type of rehabilitation, Type of material used in the restoration, Type of opposing dentition, Biofilm, Bleeding, Probing pocket depth, Bone level at diagnosis, Mechanical complications, Therapeutic approach. |
| Wang et al/2020(Wang et al., 2020) | Classification model with microbiota in identifying sites with or without suppuration | Microbial predictors |
| Zhang et al/2020(Zhang et al., 2020) | Prediction model for marginal bone loss  of dental implant in the mandible | Age, Gender, Periodontitis, Implant diameter, Implant length, Implant site, Retention selection, Cortical bone thickness, Smoking, Percent bone volume (BV/TV), Bone surface (BS), Intersection surface (i.S), Bone surface/volume ratio (BS/BV), Bone surface density (BS/TV), Trabecular pattern factor (Tb.Pf), Structure model index (SMI), Trabecular thickness (Tb.Th), Trabecular number (Tb.N), Trabecular separation (Tb.Sp) |
| Lu et al/2021(Lu et al., 2021) | A microbial prediction model for clinical suppuration | Microbial predictors |
| Mameno et al/2021(Mameno et al., 2021) | Machine learninig predictive model for peri‑implantitis | Age, sex, implant brand, functional time of the implant, history of periodontitis, PCR, number of cigarettes smoked, number of occlusal supports, cement fixation, position, KMW |
| Zhang et al/ 2021.10 | Nomogram model to predict the risk of peri-impalntitis in patients with diabetes mellitus | Age, gender, BMI, education level, smoking habbit, alcohol, tea, HbA1bc, history of periodontitis, peri-implant mucosatitis, implant site, bone graft, submerged/ non-submerged implant, interdental brush use, brushing frequency, Regular periodontal maintenance. |
| Huang et al/2022(Huang et al., 2022) | Prediction model for dental implant loss | Age, sex, smoking, blood glucose, implant sites, diameter, length, and torque |
| Oh et al/ 2023.4.8 | Deep learning-based prediction  of osseointegration for dental implant using plain radiography | Radiographic DL predictors |
| Rekawek et al/ 2023.6.19 | A Web-Based Implant Failure and Peri-implantitis  Prediction Model | Date implant placed, Patient ID, Implant ID, Date of birth, Age at placement, Age over 60 years old, Sex, Race, Tooth number, Implant location (anterior mandible, posterior mandible, anterior maxilla, or posterior maxilla), Implant diameter (mm), Implant length (mm), Implant brand, Implant reg, Implant lot, Attending, Resident, Resident ID, PGY level, OS or GPR resident, Diabetic, Diabetic status (not diabetic, diet-controlled, oral medications, insulin), HbA1c at placement after 3 months, HbA1c status, Bisphosphonate usage, Immunosuppressed, Immunosuppressive agent, Smoker, Illicit drug usage, Alcohol usage, Amount of keratinized tissue, Frequency of hygiene visits, Implant committee approval, Prior bone Ggrafting, Type of prior bone graft, Implant placed in OR or clinic setting, Peridex rinse, Preoperative antibiotics, Type of local anesthesia delivered, Amount of local anesthesia delivered (cc), Flap versus flapless surgery, Immediate implant, Mid operative x-ray taken, Drill speed (rpm), Insertional torque value, Bone graft at time of placement, Type of bone graft at time of placement, Membrane at time of placement, Correction osteotomy at placement, Soft tissue grafting at time of placement, Single or stage II, Duration of postoperative antibiotics (days), Restored Time to restoration, Screw-retained or cement-retained restoration, Type of restoration (single, splinted, overdenture, multiunit FPD), Number of implants supporting removable prosthesis (if removable), Presence of peri-implantitis, Peri-implantitis development months after placement, Treatment of peri-implantitis, Osseointegration failure, Osseointegration failure months after placement, Angulation failure, Explant, Date of explant, HbA1c at date of explant, Early failure (< 6 months), Late failure (> 6 months), Success, Date of last visit, Lost to follow-up, Prior implant failure, Postoperative pain regimen, Medication list, Diabetes medications, Antihypertensive medications, ARB sartan medications, Beta blocker medications, Calcium channel blocker medications, Diuretics, ACE inhibitors, Clonidine, PDE5 inhibitors, PPIs, SSRIs, SNRIs, Bupropion, Statins, Ezetimibe, Fenofibrate/gemfibrozil, Finasteride, Alpha-1 blockers, Methotrexate, Antipsychotics |

* Didn’t reported specific predictors in each model but listed some important predictors.

# Table *S*8. PROBAST risk of bias assessment in the 14 modeling prediction study.

| Author/Year | 1. Participants | 2. Predictor | 3. Outcome | 4. Analysis | Overall |
| --- | --- | --- | --- | --- | --- |
| Papantonopoulos et al/2015 | L | L | L | H | H |
| Sampaiofernandes et al/2017 | L | L | H | H | H |
| Papantonopoulos et al/2017 | L | L | L | H | H |
| Zhang et al/2018 | L | L | L | H | H |
| Ha et al/ 2018 | L | L | L | H | H |
| Wang et al/2020 | L | L | L | H | H |
| Zhang et al/2020 | L | L | H | H | H |
| Lu et al/2021 | L | L | L | H | H |
| Mameno et al/2021 | L | L | L | H | H |
| Zhang et al/2021 | L | L | L | L | L |
| Huang et al/2022 | L | L | L | U | U |
| Oh et al/2023 | L | L | L | H | H |
| Rekawek et al/2023 | L | L | L | H | H |

Abbreviation: L, Low; H, high; U, unclear

# Table *S*9. Answers for each signalling question in PROBAST for 14 modeling prediction studies.

| Author/Year | 1. Participants | | 2. Predictor | | | 3. Outcome | | | | | | 4. Analysis | | | | | | | | |
| --- | --- | --- | --- | --- | --- | --- | --- | --- | --- | --- | --- | --- | --- | --- | --- | --- | --- | --- | --- | --- |
|  | 1.1 | 1.2 | 2.1 | 2.2 | 2.3 | 3.1 | 3.2 | 3.3 | 3.4 | 3.5 | 3.6 | 4.1 | 4.2 | 4.3 | 4.4 | 4.5 | 4.6 | 4.7 | 4.8 | 4.9 |
| Papantonopoulos et al/2015 | Y | Y | Y | Y | Y | Y | Y | Y | Y | Y | Y | Y | Y | Y | NI | Y | Y | N | Y | Y |
| Sampaiofernandes et al/2017 | Y | Y | PY | PY | Y | Y | Y | N | PY | PY | PY | PY | Y | Y | NI | Y | / | N | N | Y |
| Papantonopoulos et al/2017 | Y | Y | Y | Y | Y | Y | Y | Y | Y | Y | Y | Y | Y | Y | NI | Y | Y | N | Y | PY |
| Zhang et al/2018 | Y | Y | Y | Y | Y | Y | Y | N | Y | Y | Y | Y | Y | Y | NI | Y | / | N | N | Y |
| Ha et al/ 2018 | Y | Y | Y | Y | Y | Y | Y | Y | Y | PY | Y | PY | Y | Y | Y | Y | Y | N | Y | Y |
| de Araújo Nobre et al/2019 | Y | Y | Y | Y | Y | Y | Y | N | Y | Y | PY | Y | Y | Y | Y | N | / | N | Y | Y |
| Wang et al/2020 | Y | Y | Y | Y | Y | Y | Y | Y | Y | Y | PY | PN | Y | Y | NI | Y | Y | N | N | Y |
| Zhang et al/2020 | Y | / | Y | Y | Y | Y | Y | Y | Y | Y | / | PN | PY | Y | Y | Y | Y | N | N | Y |
| Lu et al/2021 | Y | Y | Y | Y | Y | Y | Y | Y | Y | Y | PY | PN | Y | Y | PY | Y | Y | N | N | Y |
| Mameno et al 2021 | Y | Y | Y | Y | Y | Y | Y | Y | Y | Y | PY | Y | Y | Y | Y | PY | Y | N | Y | Y |
| Zhang et al/ 2021 | Y | Y | Y | Y | Y | Y | Y | Y | Y | PY | Y | Y | Y | Y | Y | Y | Y | Y | Y | Y |
| Huang et al 2022 | Y | Y | Y | Y | Y | Y | Y | Y | Y | Y | Y | Y | Y | Y | NI | Y | Y | Y | Y | Y |
| Oh et al/ 2023 | Y | Y | Y | Y | Y | Y | Y | Y | Y | Y | Y | PY | Y | Y | NI | Y | Y | N | Y | Y |
| Rekawek et al/ 2023 | Y | Y | Y | PY | Y | Y | PY | Y | Y | PY | Y | Y | Y | Y | Y | Y | Y | N | Y | Y |

Abbreviation: Y, yes; N, No; PY, probably yes; PN, probably no; NI, no information.

# Table *S*10. Reasons for being answered “N/PN (No/Probably No)” for signalling question in PROBAST for 14 studies.

| Papantonopoulos et al/2015 | 4.4 No information on missing data.  4.7 Lack of calibration assessment. |
| --- | --- |
| Sampaiofernandes et al/2017 | 3.3 Probing depth was included in the prediction of implant success.  4.4 No information on missing data.  4.7 Lack of calibration assessment.  4.8 Lack of internal validation. |
| Papantonopoulos et al/2017 | 4.4 No information on missing data.  4.7 Lack of calibration assessment. |
| Zhang et al/2018 | 3.3 Periodontal predictors were included in the definition of outcome.  4.4 No information on missing data.  4.7 Lack of calibration assessment.  4.8 Lack of internal validation. |
| Ha et al/ 2018 | 4.7 Lack of calibration assessment. |
| De Araújo Nobre et al/2019 | 3.3 Predictors (e.g., bleeding, probing depth) were included in the definition of outcome.  4.5 The selection of variable depended on univariable analysis.  4.7 Lack of calibration assessment. |
| Wang et al/2020 | 4.1 Only 24 implants included, events per variable (EPV) less than 10.  4.7 Lack of calibration assessment.  4.8 Lack of internal validation. |
| Zhang et al/2020 | 4.1 EPV less than 10.  4.7 Lack of calibration assessment.  4.8 Lack of internal validation |
| Lu et al/2021 | 4.1 Only 24 implants included, EPV less than 10.  4.7 Lack of calibration assessment.  4.8 Lack of internal validation. |
| Mameno et al 2021 | 4.7 Lack of calibration assessment. |
| Zhang et al/2021 | NA |
| Huang et al 2022 | NA |
| Oh et al/2023 | 4.4 No information on missing data.  4.7 Lack of calibration assessment. |
| Rekawek et al/ 2023 | 4.7 Lack of calibration assessment. |

# Table *S*11. Signalling questions for PROBAST.

| **1. Participants** | **2. Predictors** | **3. Outcome** | **4. Analysis** |
| --- | --- | --- | --- |
| **Signaling questions** | | | |
| 1.1. Were appropriate data sources used, e.g., cohort, RCT, or nested case–control study data? | 2.1. Were predictors defined and assessed in a similar way for all participants? | 3.1. Was the outcome determined appropriately? | 4.1. Were there a reasonable number of participants with the outcome? |
| 1.2. Were all inclusions and exclusions of participants appropriate? | 2.2. Were predictor assessments made without knowledge of outcome data? | 3.2. Was a prespecified or standard outcome definition used? | 4.2. Were continuous and categorical predictors handled appropriately? |
|  | 2.3. Are all predictors available at the time the model is intended to be used? | 3.3. Were predictors excluded from the outcome definition? | 4.3. Were all enrolled participants included in the analysis? |
|  |  | 3.4. Was the outcome defined and determined in a similar way for all participants? | 4.4. Were participants with missing data handled appropriately? |
|  |  | 3.5. Was the outcome determined without knowledge of predictor information? | 4.5. Was selection of predictors based on univariable analysis avoided?† |
|  |  | 3.6. Was the time interval between predictor assessment and outcome determination appropriate? | 4.6. Were complexities in the data (e.g., censoring, competing risks, sampling of control participants) accounted for appropriately? |
|  |  |  | 4.7. Were relevant model performance measures evaluated appropriately? |
|  |  |  | 4.8. Were model overfitting, underfitting, and optimism in model performance accounted for?† |
|  |  |  | 4.9. Do predictors and their assigned weights in the final model correspond to the results from the reported multivariable analysis?† |

PROBAST = Prediction model Risk Of Bias ASsessment Tool. † Development studies only.

Source: Wolff RF, Moons KGM, Riley RD, Whiting PF, Westwood M, Collins GS et al. (2019). PROBAST: A Tool to Assess the Risk of Bias and Applicability of Prediction Model Studies. Ann Intern Med 170(1):51-58.

# Table S12. Diagnosis of peri-implantits in the 2017 World Workshop on the Classification of Periodontal and Peri-Implant Diseases and Conditions.

| Diagnosis of peri‐implantitis requires: |
| --- |
| • Presence of bleeding and/or suppuration on gentle probing.  • Increased probing depth compared to previous examinations.  • Presence of bone loss beyond crestal bone level changes resulting from initial bone remodeling. |
| In the absence of previous examination data diagnosis of peri‐implantitis can be based on the combination of: |
| • Presence of bleeding and/or suppuration on gentle probing.  • Probing depths of ≥6 mm.  • Bone levels ≥3 mm apical of the most coronal portion of the intraosseous part of the implant. |
